# NADPH and Mitochondrial Quality Control as Targets for a Circadian-Based Fasting and Exercise Therapy for the Treatment of Parkinson’s Disease

**DOI:** 10.3390/cells11152416

**Published:** 2022-08-04

**Authors:** William M. Curtis, William A. Seeds, Mark P. Mattson, Patrick C. Bradshaw

**Affiliations:** 1 Department of Biomedical Sciences, Quillen College of Medicine, East Tennessee State University, Johnson City, TN 37614, USA; 2 Seeds Scientific Performance Research, Spire Institute, Geneva, OH 44041, USA; 3 Department of Neuroscience, Johns Hopkins University School of Medicine, Baltimore, MD 21205, USA

**Keywords:** Parkinson’s disease, mitochondrial quality control, NADPH, DJ-1, PINK1, Parkin, IDH1, metabolic therapy, fasting, exercise, NAD, mitophagy, mitochondrial biogenesis, circadian, nicotinamide adenine dinucleotide, nicotinamide adenine dinucleotide phosphate, coffee

## Abstract

Dysfunctional mitochondrial quality control (MQC) is implicated in the pathogenesis of Parkinson’s disease (PD). The improper selection of mitochondria for mitophagy increases reactive oxygen species (ROS) levels and lowers ATP levels. The downstream effects include oxidative damage, failure to maintain proteostasis and ion gradients, and decreased NAD^+^ and NADPH levels, resulting in insufficient energy metabolism and neurotransmitter synthesis. A ketosis-based metabolic therapy that increases the levels of (R)-3-hydroxybutyrate (BHB) may reverse the dysfunctional MQC by partially replacing glucose as an energy source, by stimulating mitophagy, and by decreasing inflammation. Fasting can potentially raise cytoplasmic NADPH levels by increasing the mitochondrial export and cytoplasmic metabolism of ketone body-derived citrate that increases flux through isocitrate dehydrogenase 1 (IDH1). NADPH is an essential cofactor for nitric oxide synthase, and the nitric oxide synthesized can diffuse into the mitochondrial matrix and react with electron transport chain-synthesized superoxide to form peroxynitrite. Excessive superoxide and peroxynitrite production can cause the opening of the mitochondrial permeability transition pore (mPTP) to depolarize the mitochondria and activate PINK1-dependent mitophagy. Both fasting and exercise increase ketogenesis and increase the cellular NAD^+^/NADH ratio, both of which are beneficial for neuronal metabolism. In addition, both fasting and exercise engage the adaptive cellular stress response signaling pathways that protect neurons against the oxidative and proteotoxic stress implicated in PD. Here, we discuss how intermittent fasting from the evening meal through to the next-day lunch together with morning exercise, when circadian NAD^+^/NADH is most oxidized, circadian NADP^+^/NADPH is most reduced, and circadian mitophagy gene expression is high, may slow the progression of PD.

## 1. A Strong Rationale for Targeting MQC in PD

Decreased MQC in PD may occur through a genetic predisposition, environmental toxins, infectious disease, or physical injury, and increases with aging. A population of damaged low-quality mitochondria generates increased amounts of ROS and produces a localized deficit of ATP, possibly resulting in the increased aggregation and decreased proteolysis of aggregation-prone proteins, such as α-synuclein [1]. This dysfunctional proteostasis may be exacerbated by increased levels of ROS, produced by the dysfunctional mitochondria, and by increased amounts of reactive nitrogen species (RNS) that exceed the amount required for healthy cellular signaling. This leads to cytoplasmic NAD^+^ and NADPH depletion and cellular oxidative damage. The increased ROS production further leads to oxidation and the loss of cardiolipin and plasmalogen phospholipids, deficits in neurotransmitter production, and compromised cellular signaling pathways. Therefore, MQC is a target for PD treatment that is upstream of dopamine and α-synuclein involvement. Bolstering the mitochondrial energy metabolism and resilience is therefore predicted to halt or slow the cascade of downstream events that follow to prevent the onset, slow the progression, and relieve the symptoms of PD, including those not addressed by dopamine-directed therapies.

Genes causally associated with PD include *PINK1*, *Parkin*, *DJ-1*, *LRRK2*, and *SNCA*. From the genetic linkage analysis and follow-up studies characterizing the function of the identified gene products, it has been shown that mitophagy is a common mechanism that is dysfunctional in these genetic forms of PD [1]. LRRK2 is associated with the interruption of mitophagy by several proposed mechanisms [2]. Although a decreased mitochondrial membrane potential has been implicated in the initiation of mitophagy at the individual organelle level [3], the exact mechanism through which a mitochondrion loses its membrane potential marking it for degradation remains incompletely known. We hypothesize that part of the mechanism that selects a specific mitochondrion for degradation uses the release of superoxide (O_2_^•^^−^) from the electron transport chain (ETC) into the matrix space of organelles as a sentinel. We have named this the superoxide sentinel hypothesis of MQC and will later describe the detailed mechanism and its implications for PD.

Braak studied PD brains and found that α-synuclein deposition was not initially observed in the substantia nigra pars compacta, where dopaminergic neurons are present [4]. PD symptoms that are not caused by the loss of dopamine or that are not treated by dopaminergic therapies include prodromal symptoms, fatigue, depression, freezing, executive dysfunction, and cognitive set switching. Consistent with this fact, noradrenergic neuronal dysfunction has recently been associated with several nonmotor PD deficits in a novel PD mouse model [5]. Targeting dopaminergic neurons with L-DOPA therapy therefore cannot reverse these other symptoms. Restoring dopamine levels does not lead to a slowing of PD progression, as evidenced by the lack of effects of L-DOPA, dopamine receptor agonists, or the inhibitors of the catabolism of dopamine or L-DOPA on delaying the progression of PD. Therefore, further studies exploring the deficits that occur upstream of dopaminergic dysfunction, such as the mechanisms behind the decreased MQC that occur in PD cells are required and could lead to a significant advance in our understanding of PD pathogenesis.

## 2. Interventional Strategies to Correct Dysfunctional MQC and Downstream Processes

The rationale for the fasting and exercise therapy proposed in Section 4 below to treat PD is based on the clinical experience of the primary author, who has used the therapy to decrease his PD symptoms. Instead of focusing on PD interventional therapies that directly target MQC, as has been elaborated in the past [6], it is proposed that therapies that restore the cytoplasmic [NAD^+^]/[NADH] and [NADP^+^]/[NADPH] ratios will be more beneficial and target events upstream of MQC to directly improve cellular bioenergetics, metabolism, and antioxidant defense, and stimulate signaling pathways leading to the improvement of MQC. For example, increasing the NAD^+^/NADH ratio has been shown to activate AMPK [7], which stimulates mitophagy [8]. As another example, increased NADPH has been shown to inhibit histone deacetylase 3 (HDAC3) [9], and HDAC3 inhibition has been shown to increase autophagy [10] and elevate Nrf2 transcriptional activity and the antioxidant response [11]. In contrast to its effect on HDAC3, NADPH stimulates HDAC1 and HDAC2 activity [12]. Increased mitochondrial NADPH levels in PD neural cells may also directly lead to increased ETC complex I activity, as complex I becomes destabilized without NADPH binding to its 39 kD NDUFA9 subunit [13,14].

### 2.1. NADPH Is Required for Proper MQC

**Figure 1:**
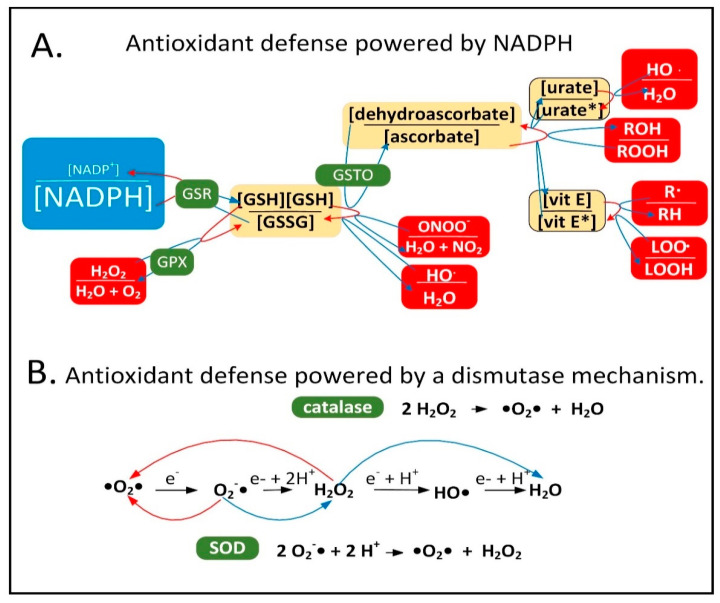
Cellular antioxidants that use NADPH for reduction compared to those that use a dismutase mechanism. (**A**) NADPH provides electrons to the antioxidant system to detoxify ROS/RNS. Abbreviations: oxidized glutathione disulfide (GSSG), hydrogen peroxide (H_2_O_2_), dehydroascorbate (oxidized form of vitamin C), peroxynitrite (ONOO^−^), hydroxyl radical (HO^•^), oxidized urate radical (urate*), oxidized vitamin E radical (vit E*), alkyl peroxide (ROOH), alkyl free radical (R^•^), lipid peroxide radical (LOO^•^) glutathione reductase (GSR), glutathione peroxidase (GPX), dehydroascorbate reductase (GSTO). (**B**) The dismutase mechanism of SOD and catalase uses two reactants (either two O_2_^•−^ or two H_2_O_2_ molecules) and leads to the reduction of one and the oxidation of the other. Abbreviation: superoxide dismutase (SOD).

Neuroinflammation is induced in the PD brain and proinflammatory cytokines are secreted by astrocytes and microglia leading to the expression of inducible nitric oxide synthase (iNOS) in these cell types [15]. iNOS synthesizes nitric oxide (^•^NO), which reacts with O_2_^•−^ to form peroxynitrite (ONOO^−^). It is hypothesized that low-quality damaged mitochondria that need to be degraded through mitophagy synthesize excess O_2_^•−^, which performs a sentinel function. ONOO^−^ is toxic and can be further metabolized into other forms of ROS/RNS that the mitochondrion is not well-equipped to detoxify. NADPH provides electrons to many of the mitochondrial antioxidants that detoxify ROS/RNS, including glutathione and thioredoxin, that further donate electrons to peroxiredoxins, glutaredoxins, vitamin C, vitamin E, and R-lipoic acid (Figure 1A).

When ROS/RNS levels begin to increase in the mitochondrial matrix, NADPH levels begin to decline due to its oxidation for the reduction of cellular antioxidants. This decline stimulates inner membrane nicotinamide nucleotide transhydrogenase (NNT) to use NADP^+^ and reducing equivalents from NADH to synthesize and restore NADPH levels. This reaction is coupled with the transport of a proton down its concentration gradient into the matrix space simultaneously decreasing the mitochondrial membrane potential. When ROS/RNS levels increase greatly NADPH becomes oxidized beyond a certain threshold leading to the oxidation of the entire NADPH antioxidant system including glutathione and thioredoxin, which results in the oxidation of cysteine residues in the matrix space and inner membrane proteins. This has been shown to cause mPTP opening, fully depolarizing the inner mitochondrial membrane to initiate a signaling cascade activating PINK1 protein kinase and Parkin ubiquitin ligase leading to mitophagy of the organelle. The role of the mPTP as an initiating event in mitophagy was first proposed roughly 25 years ago [16,17]. The antioxidant enzymes superoxide dismutase (SOD) and catalase both use a dismutase mechanism to detoxify ROS that is independent of NADPH (Figure 1B). Catalase is present in peroxisomes and not normally found in mitochondria in mammalian tissues, although exceptions exist [18,19].

### 2.2. Mechanisms of Mitochondrial Protein Turnover

Studies determining the lifespan of proteins in mitochondria of rodent brain found that the average protein lifespan was roughly three to four weeks [20,21], and this was slightly longer than in other postmitotic tissues where mitochondrial proteins have an average lifespan of nine days to two weeks [20,22,23]. However, many mitochondrial inner membrane proteins associated with cristae, especially the proteins of ETC complex III and the ATP synthase (complex V), have lifespans longer than four months. These are called long-lived proteins (LLPs) [24,25]. The presence of these LLPs supports the theory that most mitophagy events do not occur randomly regardless of the mitochondrial membrane potential. The data support a model where mitochondria undergo fusion and fission, and it is the fissioned mitochondria with a decreased membrane potential that are specifically selected for mitophagy [26]. There were few LLPs in the outer mitochondrial membrane suggesting that the outer membrane proteins are turned over by the proteasome or other cytoplasmic or intermembrane space proteases at a faster rate than entire organelles. Due to decreased rates of protein degradation with aging, proteins in aged brains had on average a 20% longer half-life than those from young brains, but some mitochondrial proteins linked to neurodegeneration showed a shorter half-life in aged brains [27]. The relatively short lifespan of cellular proteins contrasts with the long lifespan of dopaminergic and most other neurons, which are as old as the organism.

To maintain mitochondrial mass due to the ongoing mitophagy of organelles, there is an ongoing synthesis of mitochondrial proteins, DNA, and phospholipids. This process of mitochondrial biogenesis is largely triggered by the PGC-1 family of transcriptional co-activators including PGC-1α, PGC-1ß, and PPRC1 (PRC), and several transcription factors including NRF1 (nuclear respiratory factor 1), NRF2 (dimer of GABPA and GABPB1), ESRRA (ERR-α), ESRRG (ERR-ɣ), and NFE2L2 (Nrf2). Mitophagy in a dopaminergic neuron is complicated by the fact that the neurons have arbors of axons and dendrites that, when added together total roughly 4.1 m. Mitochondria are transported from the cell bodies down the axons to synaptic terminals to supply ATP locally at the synapses. Most mitophagy occurs in cell bodies, although some occurs in axons. Therefore, dysfunctional mitochondria at synapses are most frequently transported back to the cell body for degradation. It is estimated that there are two million mitochondria in a single dopaminergic neuron [28]. Therefore, the key to MQC in neurons is not only the ability to select the dysfunctional mitochondria for degradation, but also the ability to transport the dysfunctional mitochondria to the mitophagy machinery where the degradation takes place. Both processes are often dysfunctional in PD [29].

The regulation of MQC can be complex and for this reason the mechanisms through which individual organelles are selected for mitophagy are not that well characterized, although there is strong evidence that decreased mitochondrial membrane potential plays a role. There are several different proteolytic systems present in mitochondria including proteases inside the mitochondrial matrix (LONP1, CLPP/CLPX, PITRM1, XPNPEP3, and MIPEP), in the inner membrane (AFG3L2, SPG7, IMMP1L, IMMP2L, OMA1, PARL, YME1L1, and PMPCA/PMPCB), in the intermembrane space (HTRA2 and LACTB), and in the outer membrane (USP30) [30] that degrade proteins independently of mitophagy. Mitochondrial membranes that contain soluble proteins can also bud off from the mitochondria, and these are called mitochondrial-derived vesicles (MDVs). PINK1 and Parkin may be involved in the formation of MDVs. The cell may also possess a non-selective form of autophagy that slowly degrades the mitochondria indiscriminately [31]. 

### 2.3. DJ-1 Is Regulated by Cytoplasmic NADPH and Glutathione and Controls Nrf2 Activation in Astrocytes

**Figure 2:**
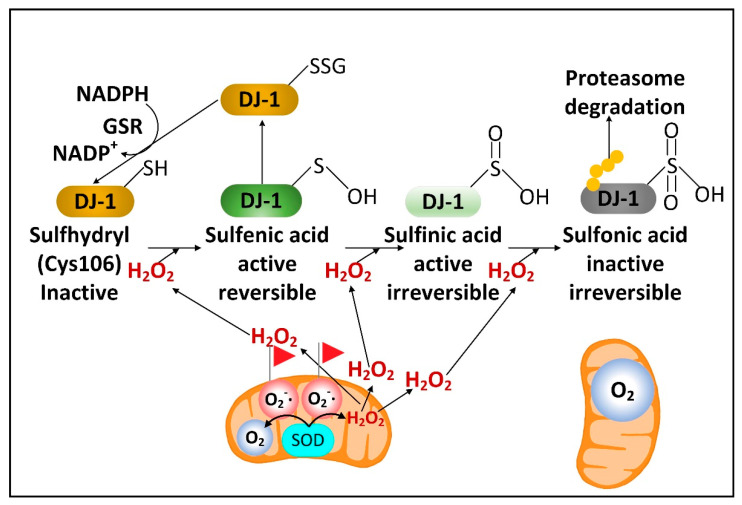
H_2_O_2_ activates DJ-1. O_2_^•−^ released into the mitochondrial matrix space is converted into O_2_ and H_2_O_2_ by SOD2. Mitochondrial H_2_O_2_ can be transported into the cytoplasm where moderate levels activate DJ-1, but high levels lead to DJ-1 inactivation and degradation.

There is a rare human DJ-1 mutant variant linked with recessive PD. There is evidence that preventing the decline of the cytoplasmic NADPH levels in PD will preserve the function of DJ-1, a cytoplasmic redox-sensitive chaperone protein sensor of H_2_O_2_ levels and an activator of Nrf2. DJ-1 protein is present at higher levels in human astrocytes than neurons [32]. But unexpectedly, DJ-1 mRNA is found at higher levels in neurons than astrocytes. This suggests complex post-transcriptional regulation of DJ-1 levels and that DJ-1 could be degraded in response to high levels of neuronal H_2_O_2_. Nrf2 is a master transcriptional regulator that binds to antioxidant response elements (AREs) in the promoters of genes to induce transcription. In the brain, like DJ-1, Nrf2 is present at much higher levels in astrocytes than neurons. Complexly, DJ-1 is activated by H_2_O_2_ oxidation of cysteine 106 sulfhydryl, but too much oxidation converts this cysteine sulfhydryl into a sulfonic acid, permanently deactivating the protein and marking it for ubiquitination and proteasome degradation. NADPH and glutathione are critical in preserving the active sulfenic acid and sulfinic acid forms of DJ-1 and preventing them from being further oxidized to the inactive sulfonic acid form (Figure 2).

**Figure 3:**
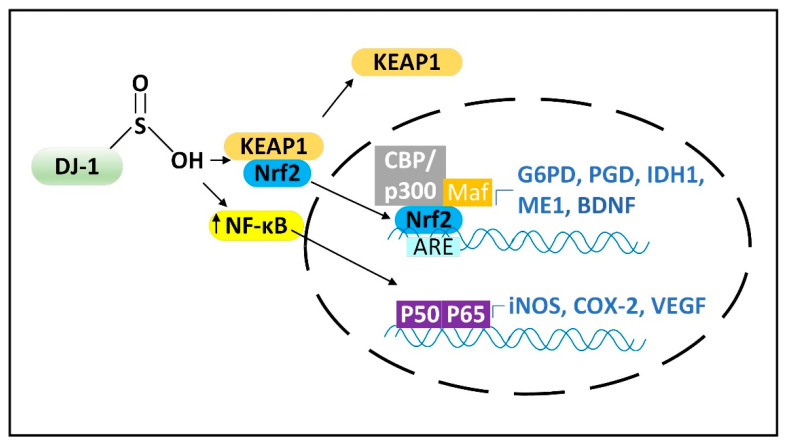
DJ-1 activates Nrf2 and NF-κB. Active forms of DJ-1 where a cysteine is oxidized to a sulfenic acid (not shown) or sulfinic acid activate Nrf2 and NF-κB. Abbreviations: Glucose-6-phosphate dehydrogenase (G6PD), 6-phosphogluconate dehydrogenase (PGD), brain-derived neurotrophic factor (BDNF), inducible nitric oxide synthase (iNOS), cyclooxygenase-2 (COX-2), vascular endothelial growth factor (VEGF).

In the active form DJ-1 releases Nrf2 from its inhibitor Keap1 to induce Nrf2 nuclear translocation to stimulate gene expression. DJ-1 is localized both to the cytoplasm and to mitochondria and can prevent alpha-synuclein fibrillization and mitochondrial fission induced by oxidative stress [33]. Due to their mitochondrial localization, DJ-1, PINK1, and Parkin are often described as functioning together to stimulate mitophagy. Nrf2 can bind to the promoters and induce the expression of four NADPH synthesizing enzymes, including IDH1, ME1, and the two NADPH-generating pentose phosphate pathway (PPP) enzymes, glucose-6-phosphate dehydrogenase (G6PD) and 6-phosphogluconate dehydrogenase (PGD) [34], as well as brain-derived neurotrophic factor (BDNF) [35]. Loss of DJ-1 decreased the mRNA stability, protein levels, and activity of activating transcription factor 4 (ATF4) in neurons. DJ-1 can also induce the nuclear translocation of NF-κB, which induces the expression of iNOS (Figure 3) [36] and the mitochondrial uncoupling proteins UCP4 and UCP5 [37,38]. Increased iNOS levels lead to increased levels of cytoplasmic ^•^NO, which can diffuse into mitochondria. If the mitochondrion is damaged and mitochondrial ETC is inefficient, there will be high levels of the matrix space O_2_^•−^ that binds to the ^•^NO, forming high levels of damaging ONOO^−^.

### 2.4. Fasting Increases the Hepatic Levels of ATF4 to Increase NADPH, FGF21, and Parkin

ATF4 is a transcriptional regulator involved in many signaling pathways that lead to either protective or degenerative outcomes depending upon the specific condition. ATF4 can induce the expression of genes that increase the levels of ROS to activate Nrf2, a binding partner for ATF4 [39]. Nrf2 activation prevents the ATF4-mediated induction of the proapoptotic gene CHOP [40]. Low-level or transient activation of ATF4 appears to be cytoprotective [41], while chronic high-level ATF4 activation can lead to cell death [42]. ATF4 can be activated by several mechanisms including the integrated stress response (ISR) [43], mitochondrial dysfunction [44], and insulin receptor signaling/mTORC1 kinase activation [45]. ATF4 is canonically activated by the selective translation of its mRNA [46]. There are at least four sets of ATF4 target genes that mediate cytoprotection. These are (1) NADPH-synthesizing enzymes, including PPP enzymes [47,48] and enzymes involved in serine catabolism and one-carbon metabolism [49]; (2) the cystine transporter SLC7A11 (also called xCT) that leads to increased glutathione synthesis [45]; (3) fibroblast growth factor 21 (FGF21), an anti-aging hormone [50,51] and mitokine [52] released predominately from the liver during fasting and exercise [53], which stimulates energy metabolism [54]; and (4) Parkin, which stimulates mitophagy [55]. Mouse hepatic ATF4 levels are positively correlated with lifespan in five long-lived genetic strains or dietary conditions, including calorie restriction (CR) [56]. Therefore, ATF4 may also mediate some of the beneficial effects of fasting on MQC in the brain, although increased neuronal ATF4 levels can also induce neurotoxicity. ATF4 expression fluctuates in a circadian manner [57], and ATF4 controls the expression of many of the core circadian clock genes [58]. Therefore, the fluctuating circadian expression of ATF4 may be a key to its cytoprotective effects. More studies are required to determine whether fasting increases cytoprotective ATF4 transcriptional activity in neurons, astrocytes, or microglia, or whether fasting and ATF4-dependent neuroprotection is primarily a result of the hepatic release of blood–brain barrier (BBB)-permeable FGF21. 

### 2.5. Fasting Activates Glutamate Dehydrogenase to Increase the Levels of the Anti-Aging Metabolite αkg

In hepatocytes and pancreatic beta-cells, fasting or CR has been shown to decrease the levels of mitochondrial sirtuin SIRT4 [59,60]. SIRT4 ADP-ribosylates and inhibits mitochondrial glutamate dehydrogenase, an enzyme which deaminates glutamate into αkg and ammonia. Therefore, under well-fed conditions when glutamate dehydrogenase activity is low, glutamate and glutamine levels increase leading to the activation of mTORC1. Under fasting conditions when glutamate dehydrogenase activity is high, αkg levels rise [60]. This regulation might be partly responsible for the diurnal fluctuations in skeletal muscle αkg levels [61]. Supplementing with αkg has been shown to increase the lifespan of *C. elegans* [62], *Drosophila* [63], and mice [64], while plasma αkg levels decline substantially with aging in mammals [65]. Brain αkg and citrate levels declined in aged rats. This decline as well as the decreased cerebral blood flow that occurred with aging were prevented by CR [66].

The protective anti-aging effects of αkg appear to be due to several mechanisms including (1) the stimulation of prolyl hydroxylase 2 (PHD2) activity to inhibit phospho-AKT activity of the insulin receptor/AKT/mTOR signaling pathway [67], (2) the activation of AMPK [63], (3) the decrease in proinflammatory senescence-associated secretory phenotype (SASP) of senescent cells [64], (4) the stimulation of KDM2-7 histone demethylases to decrease histone H3K9me3 and H3K27me3 [68], and (5) the stimulation of TET1-3 hydroxylases involved in DNA demethylation. The stimulation of histone and DNA demethylation prevents heterochromatin formation and maintains cytoprotective gene expression. Supplementation with αkg was shown to protect mouse motor function and ETC complex I activity and decrease alpha-synuclein accumulation following MPTP treatment in a mouse model of PD [69]. Exercise also increased plasma levels of αkg [70]. Unlike the deficiency of PGD of the oxidative PPP, which led to increased αkg levels in T cells, regulatory T cell (T_reg_) dysfunction, fetal inflammatory disorder, and an improved anti-tumor response [71], the deficiency of transketolase of the non-oxidative PPP led to decreased αkg levels in T cells, partly by stimulating the reductive carboxylation of αkg to isocitrate [72]. The reduced αkg levels led to DNA hypermethylation associated with T_reg_ dysfunction and lethal autoimmunity.

### 2.6. Fasting Decreases the Cytoplasmic [NADP^+^]/[NADPH] Ratio in Liver, but Its Effects on Neural Cell [NADP^+^]/[NADPH] Ratios Are Not Yet Known

Flux through enzymes that synthesize cytoplasmic NADPH changes between the well-fed and fasted/ketotic states. In the well-fed state when blood and tissue glucose levels are high, the PPP is primarily used in most tissues for the synthesis of NADPH. However, after 24 h of fasting when glycogen levels are depleted human blood glucose levels start to decline. On average, blood glucose levels rose by roughly 8% following a meal and returned to baseline three to four hours after the mealtime [73]. Blood glucose levels in human adults were shown to have declined by 6% following a 24 h fast [74] and by 25% following a 72 h fast and remained stable at that level for weeks of starvation [75]. In comparison, adult human blood BHB levels rose to approximately 0.4 mM within 16–24 h of fasting, to 1 mM after 48 h of fasting, and to 2 mM after 72 h of fasting [76]. Due to the fasting-induced decline in blood and cellular glucose levels and cellular PPP flux, the PPP, together with IDH1 and ME1 (and serine catabolism in the liver), are likely used for cytoplasmic NADPH synthesis in the fasted state. 

Any fasting and BHB-induced boost in cytoplasmic NADPH synthesis in neural cells may be limited by the moderately low activity of IDH1 in most neural cell types [77] and by the lack of expression of ME1 in neurons as shown by studies of bovine brain [78]. As a minor source of cytoplasmic NADPH in most tissues, reducing equivalents from mitochondrial NADPH, such as those generated by NNT activity or serine and glycine catabolism, can be shuttled to the cytoplasm via the reductive decarboxylation of αkg to isocitrate catalyzed by the Krebs cycle enzyme isocitrate dehydrogenase 2 (IDH2). The IDH2-catalyzed reductive carboxylation reaction concurrently oxidizes NADPH. Next, mitochondrial isocitrate is exported to the cytoplasm, followed by the cytoplasmic IDH1-catalyzed reduction of NADP^+^ to NADPH linked to the oxidation of isocitrate to αkg. The decreased level of serine found in the PD subject plasma [79] may be a result of its increased catabolism fueling NADPH synthesis for antioxidant defense as well as its decreased synthesis caused by lowered levels of DJ-1 and ATF4 as mentioned earlier.

Due to NADPH-mediated feedback inhibition of G6PD [80], the rate-limiting enzyme of the PPP, G6PD activity and PPP flux only occur at a fraction of their maximal activity under normal conditions in vivo. This strong product inhibition of G6PD functions to buffer and stabilize cytoplasmic NADPH levels. Surprisingly, hepatocytes have little to no PPP activity and generate most cytoplasmic NADPH through serine catabolism [81]. In the liver in the fasted state fatty acids are imported into hepatocyte and transported to the mitochondrial matrix for beta-oxidation and ketone body synthesis. As part of the citric acid cycle some of the fatty acid-derived mitochondrial acetyl-CoA condenses with oxaloacetate to form citrate. Some citrate is exported to the cytoplasm where it is metabolized into downstream intermediates such as isocitrate and malate, which are metabolized by IDH1 and ME1, respectively, for the synthesis of NADPH. This metabolism is likely responsible for the lower hepatic cytoplasmic [NADP^+^]/[NADPH] ratio in the fasted state compared to the well-fed state. For example, a 48 h fast in rats led to an 84% decrease in the hepatic cytoplasmic [NADP^+^]/[NADPH] ratio [82]. However, after five days of fasting, the hepatic NADPH levels returned to baseline and markers of oxidative stress increased [83]. Thus, longer-term fasting may not yield the same potent metabolic benefits as 48 h fasting. 

Ketone ester consumption led to a 62% decrease in the cytoplasmic [NADP^+^]/[NADPH] ratio in the cerebral cortex, but no change occurred in the hippocampus of APP/PS1 AD model mice [84]. The reduced coenzyme ratio in the cortex following ketone ester consumption suggests that glucose levels do not need to decline for BHB metabolism to positively affect cytoplasmic nucleotide coenzyme ratios, and that fasting for less than 24 h in humans may impart beneficial metabolic effects through BHB-mediated alterations in nucleotide coenzyme ratios. However, it is not yet known whether fasting can decrease the cytoplasmic [NADP^+^]/[NADPH] ratio in tissues other than liver that contain an active PPP. 

Fasting increases mitochondrial NADPH synthesis due to increased mitochondrial SIRT3 expression, which leads to the deacetylation and activation of the NADPH-synthesizing enzyme IDH2 [85]. The activity of mitochondrial NAD^+^ kinase NADK2 is also inhibited by lysine acetylation. NADK2 is deacetylated by SIRT3 [86] likely contributing to a fasting-induced increase in the mitochondrial matrix NADPH levels. However, at the date of the publication of this review, direct evidence for the SIRT3-mediated stimulation of NADK2 activity in vivo in response to fasting or CR was still lacking.

### 2.7. Fasting May Improve PD Symptoms by Decreasing the Number of Senescent Astrocytes and Microglia without Affecting Substantia Nigra Mitochondrial DNA Deletion Levels

There is now substantial evidence suggesting that the accumulation of senescent cells plays a role in aging and aging-related disorders. Studies with mice demonstrated the accumulation of senescent astrocytes and microglia following the injection of alpha-synuclein pre-formed fibrils into the brain. MPP^+^ was also shown to stimulate cellular senescence [87]. Excitingly, paraquat-induced PD symptoms in mice were mitigated by killing senescent cells [88]. Cellular senescence in vitro is typically characterized by increased mitochondrial fusion and decreased mitophagy leading to increased mitochondrial mass and increased ROS levels [89]. 

One mechanism through which cellular senescence may be induced is by the release of mitochondrial DNA (mtDNA) into the cytoplasm by the opening of the mPTP activating the cyclic GMP-AMP synthase (cGAS) and stimulator of the interferon genes (STING) pathway [90]. The mPTP has a pore diameter of roughly 3 nm. Thus, linear double-strand mtDNA with a diameter of 2.3 nm appears to be threaded through the pore and released into the cytoplasm. Mitochondrial FEN1 nuclease has been shown to be activated by oxidative stress and to hydrolyze oxidized mtDNA into 500–650 base pair fragments released through the mPTP into the intermembrane space [91]. Mitochondrial outer membrane VDAC pores can also transport solutes such as double-strand DNA with a diameter less than 3 nm between the intermembrane space and the cytoplasm [92]. Although there are currently little data to support this mechanism, mitochondrial depolarization activating calcium-independent phospholipase A2-γ (PNPLA8) [93] may lead to the hydrolysis of mitochondrial inner membrane phospholipids to release larger mtDNA fragments. Current data suggest that PNPLA8 activated by mitochondrial depolarization hydrolyzes outer membrane phospholipids for the release of cytochrome c into the cytoplasm. MtDNA fragments of sizes up to 1000 base pairs [94,95] have been shown to be released and trigger inflammation by activating the NLRP3 inflammasome, which can contribute to cellular senescence. Supplementation with nicotinamide riboside, an NAD^+^ precursor, was recently demonstrated to increase mitophagy, decreasing NLRP3 inflammasome activation and cGAS-STING activity to decrease neuroinflammation and cellular senescence in an APP/PS1 mouse model of AD [96].

The substantia nigra in both aged PD subjects and healthy aged controls is characterized by high amounts of mtDNA deletions leading to mitochondrial ETC dysfunction [97,98]. Subjects without PD, however, compensate for these mtDNA deletions by increasing mitochondrial biogenesis [99], perhaps through a PINK1–Parkin–PGC-1α-dependent pathway. This topic was recently reviewed [100]. Homozygous POLG^D257A^/^D257A^ mtDNA mutator mice have been shown to have increased levels of mtDNA deletions and increased amounts of senescent cells possessing a unique senescence-associated secretory phenotype where proinflammatory cytokines are not secreted [101]. Increased levels of apoptosis likely contribute to the premature aging phenotype of this mouse strain [102]. Studies using control and heterozygous and homozygous mtDNA mutator mice have shown a strong correlation between the presence of mtDNA deletions and the premature aging phenotype. However, mtDNA deletor mice transgenic for a mutant form of the mitochondrial TWNK helicase do not show a premature aging phenotype [103]. Heterozygous mtDNA mutator mice have moderately high levels of mtDNA point mutations, but do not contain many mtDNA deletions or exhibit the premature aging phenotype [104]. 

CR did not reduce the mtDNA deletion load or extend the short lifespan of homozygous mtDNA mutator mice [105]. CR also did not reduce mtDNA deletion load in aged rhesus macaque skeletal muscle [106], but it did delay sarcopenia [107]. This suggests that CR inhibited muscle fiber death downstream of mtDNA deletions not by selectively increasing mitophagy of organelles containing mtDNA deletions, but likely by increasing mitochondrial biogenesis to compensate for the mitochondrial ETC dysfunction caused by the mtDNA deletions [108].

The decreased mitophagy rate in PD dopaminergic neurons may be mechanistically linked with the decreased mitochondrial biogenesis rate to maintain stable levels of mtDNA, as Parkin-deficient iPSC-derived neurons that show deceased mitophagy also showed decreased PGC-1α levels with decreased mitochondrial biogenesis. It is not yet known whether the accumulation of mtDNA deletions leads to increased release of mtDNA into the cytoplasm resulting in cellular senescence in the substantia nigra that stimulates PD onset and progression. Data from mice suggest a two-hit model where mtDNA mutator mice also require *Parkin* knockout that decreases mitophagy and mitochondrial biogenesis to release mtDNA into the cytoplasm to induce the cGAS-STING pathway and the proinflammatory senescence phenotype. Studies quantifying cellular senescence in subjects with mtDNA deletion syndromes such as Kearns–Sayre syndrome (KSS), Pearson syndrome, or progressive external ophthalmoplegia (PEO) may help to clarify this relationship in humans. Aged mice contain a 10-fold lower level of mtDNA deletions in the substantia nigra than that present in aged humans. In the mouse substantia nigra, similar to that of healthy humans, mitochondrial protective pathways such as mitochondrial biogenesis appear to be activated in response to increased levels of mtDNA deletions in an attempt to compensate for the ETC dysfunction [109], possibly explaining why 12–14-month-old homozygous mtDNA mutator mice that contained high levels of mtDNA deletions did not show a loss of dopamine or dopaminergic neurons although striatal levels of tyrosine hydroxylase, the rate-limiting enzyme of catecholamine synthesis, were decreased and motor function was moderately impaired [110].

### 2.8. A Requirement for Fasting in Dietary Restriction-Induced Longevity

Recent studies using mice have shown that dietary restriction did not extend longevity when the mice were fed many small meals without an extended period of fasting [111], and that 13 h daily fasting extended the mouse lifespan even when the mice were not calorie restricted [112]. Insulin signaling leads to the phosphorylation and exclusion from the nucleus of the pro-longevity transcription factor FOXO3A. When insulin levels decline during fasting, FOXO3A is not phosphorylated, leading to its translocation into the nucleus to mediate the changes in gene expression required for the lifespan extension that occurs due to dietary restriction/intermittent fasting [113]. Therefore, it appears likely that BHB mediates some of its neuroprotective and disease-modifying effects through stimulating FOXO3A-dependent transcription. Consistent with this hypothesis, the FOXO3A homolog DAF-16 is required for BHB-mediated lifespan extension in *C. elegans* [114]. 

### 2.9. Evidence That Combining Fasting and Exercise Is Neuroprotective

For an extensive discussion of how fasting and exercise improve neural function, the following review article is highly recommended [115]. The authors provide evidence that intermittent metabolic switching (IMS) between glucose and ketone body metabolism maintains brain function at a high level. Exercise, which stimulates glucose consumption and glycogen breakdown, facilitates the switch from glucose to ketone body metabolism. Although exercise by itself has been shown to have little effect on the improvement of the gait in PD patients [116,117], exercise has been shown to improve aspects of memory and decrease depression and anxiety in both humans and animal models [118]. Treadmill exercise also improved the motor symptoms of MPTP-treated mice [119]. Importantly, studies of mice that combined dietary restriction with voluntary wheel running demonstrated greater increases in hippocampal dendritic spine density than either therapy alone [120]. In addition, neurogenesis in the dentate gyrus was stimulated by both fasting [121] and exercise [122]. At the molecular level both fasting [123] and exercise [124] stimulated the activity of neuronal CREB and NF-κB transcription factors leading to the increased expression of brain-derived neurotrophic factor (BDNF) to protect neurons [125]. BHB by itself can also increase BDNF production by neurons [126,127], but only when glucose levels are low [128].

Like fasting, exercise decreases neuroinflammation [129], which may be partly due to BHB binding the HCAR2 receptor and the NLRP3 inflammasome blocking proinflammatory signaling [130]. However, BHB administration was unable to block the NLRP3 inflammasome activated by alpha-synuclein in microglia. Encouragingly, a two-year trial of cognitive training, diet, and exercise led to improved cognition in AD patients [131]. This suggests that it may also be possible to use combined diet and exercise therapy to slow the progression of PD. Studies on the effects of a ketogenic diet by itself, without exercise therapy, on PD subject symptoms have recently been reviewed [132]. Unlike the results from mouse models of PD where the ketogenic diet has shown moderate protective effects, the clinical results from PD subjects showed that the diet did not affect motor symptoms and only showed slight improvements in the non-motor symptoms [133].

### 2.10. Exercise and Its Effects on PD Brain and Muscles

Exercise is categorized into aerobic (endurance) training or anaerobic (resistance) training. The intensity and volume or duration of the workout determines whether enough O_2_ is transported to the skeletal muscles for aerobic metabolism to occur. Aerobic and anaerobic training are both important for the release of myokines to improve systemic homeostasis and decrease inflammation. Aerobic exercise was shown to increase the levels of the myokines VEGF-A, SPARC, sestrin, SDF-1, irisin, IL-6, IL-15, BAIBA, and apelin, while anaerobic exercise increased the levels of VEGF-A, irisin, IL-6, IL-15, IGF-1, decorin, and BMP-7. Both types of exercise decreased the myostatin secretion. With aging, the secretion of VEGF-A, SPARC, sestrin, SDF-1, irisin, IL-15, IGF-1, decorin, BMP-7, BAIBA, and apelin decreased, while that of IL-6 and myostatin increased [134]. Therefore, exercise therapies can now be tailored to specific aging-related diseases, such as PD. Examples of different types of exercise that have been shown to be beneficial for PD include resistance training, dance, yoga, and virtual reality training [135].

Different types of exercise can have different molecular effects on neural and skeletal muscle gene expression. Aerobic endurance exercise has historically been shown to have the ability to increase nuclear and mitochondrial gene expression, thus increasing skeletal muscle metabolic efficiency. Maintaining efficient mitochondrial coupling, the amount of ATP synthesized per O_2_ reduced to H_2_O by oxidative phosphorylation, relies on sustained rates of mitophagy and mitochondrial biogenesis. These processes are impaired in several tissues of PD subjects, leading to complex I and complex IV deficits in the substantia nigra and skeletal muscles [136,137,138]. However, one study did not find mitochondrial complex I dysfunction in PD subject skeletal muscles, platelets, or brain regions outside the substantia nigra [139]. Different muscle fiber types also rely on oxidative phosphorylation to different extents. Fatigue and impaired exercise capacity in PD subjects are likely attributable to mitochondrial dysfunction. Aerobic endurance exercise increases mitochondrial biogenesis in the brain and skeletal muscles by increasing the expression and activities of PGC-1α, its binding partner NRF1, and their transcriptional target mitochondrial transcription factor A (TFAM). The increased mitochondrial biogenesis and turnover increase mitochondrial and myofiber quality [140]. Aerobic endurance exercise also increased the ratio of mitochondrial fusion to fission in skeletal muscles favoring a more tubular mitochondrial network associated with enhanced ETC activity. Aerobic training improves peak O_2_ uptake (VO_2_peak) and increases skeletal muscle nuclear and mitochondrial gene expression to increase mitochondrial enzyme activities, such as that of the TCA cycle enzyme citrate synthase, used as a marker of mitochondrial mass and that of mitochondrial ETC complexes III, IV, and V [140].

The transcription of autophagy- and mitophagy-related genes is controlled by transcription factor EB (TFEB), a master regulator of lysosomal biogenesis. Endurance training increased TFEB expression and transcriptional activity in a PGC-1α-dependent manner. Thus, following exercise TFEB increases autophagic and mitophagic flux to turnover mitochondria and other cellular components that were damaged by the increased ROS produced during the exercise event [141]. In the hypoxic ischemic encephalopathy rat model, endurance training preserved hippocampal and cerebral cortical mitochondrial membrane integrity to lower cytoplasmic and nuclear levels of the mitochondrial apoptosis-inducing proteins AIF and cytochrome c, as well as cleaved caspase-3. The antiapoptotic impact of exercise training improved motor function, learning, and memory suggesting that exercise training stabilizes mitochondrial function to protect against cell death in neurodegenerative disease [142]. 

More recently, moderate repetition resistance training has also been shown to improve oxidative capacity, and this was shown under specific conditions to be more effective than aerobic endurance training in improving mitochondrial coupling efficiency and inducing qualitative changes in motor performance. Resistance training increased skeletal muscle complex I-mediated respiratory activity and the levels of many subunits of oxidative phosphorylation complexes I-V, as well as the levels of mitochondrial fusion proteins [143] and nicotinamide phosphoribosyl transferase (NAMPT), the rate-limiting step in NAD^+^ salvage synthesis [144]. Resistance training also improved the coupling efficiency in skeletal muscle mitochondria and increased the ratio of oxidative capacity to mitochondrial mass [145]. Resistance training, when performed after endurance training, amplified the induction of PGC-1α and PPAR-β/δ and led to an increased amount of mitochondrial biogenesis [145].

High-intensity interval training has also recently been demonstrated to result in endurance-like adaptations in cellular efficiency. Matched-work continuous and intermittent exercise both led to acute and chronically improved insulin sensitivity and reduced glycogen utilization and lactate production [146]. High-intensity interval training, with the increased recruitment of type-II fibers, led to increased AMPK activity and PGC-1α expression in response to increased exercise intensity and cellular energy demand [147]. Further evaluation of low-volume, high-intensity interval training indicated improved mitochondrial protein content and enzyme activities. The increased mitochondrial protein content (~25%) was consistent with that reported previously following endurance training or higher intensities of high-intensity interval training [148]. The increased aerobic capacity matched the increased COX I (MT-CO1) and COX IV (COX4I1) complex IV subunit levels. There were also increased levels of pyruvate dehydrogenase kinase 2 (PDK2). PDK2 phosphorylates and inhibits the pyruvate dehydrogenase complex (PDC) resulting in a decreased reliance on glycolysis and increased reliance on fatty acid beta-oxidation [148].

A global gene expression analysis was performed on autopsied human hippocampus to determine the genes where the expression was altered by neurodegeneration and restored in aged and disease-matched subjects who were known to have engaged in moderate physical activity prior to death. Genes associated with synaptic functions were repressed in the PD subjects and these were increased with physical activity. Specific genes that followed this expression pattern include synaptophysin, synaptotagmin-1, vesicle-associated membrane proteins, syntaxin, and complexin-I. The transcription factor Nrf1 (NFE2L1), which is involved in the expression of proteasomal genes, was also identified as following this pattern. Genes involved in transcriptional repression and negative growth regulation were increased in PD subjects and decreased with exercise [149]. 

In PD subjects, autonomic dysfunction, fatigue, and loss of exercise capacity can sometimes precede the progression of motor dysfunction by more than a decade [150,151]. In these subjects the appropriate autonomic adjustments may not accompany exercise, which can lead to inadequate hemodynamic responses, a failure to meet the metabolic demands of skeletal muscle, and exercise intolerance [152]. High-intensity interval training modulates the autonomic nervous system and heart rate by decreasing the activity of the parasympathetic system and increasing the activity of the sympathetic system [153]. High-intensity interval training also increases neural adaptation leading to decreased sympathetic and increased parasympathetic stimulation at rest. After just two weeks of training there were increased beneficial responses in cardiac autonomic control [153] that would likely benefit PD patients.

Many PD subjects have difficulty engaging in any type of physical exercise due to their dysfunctional gait. Metabolically beneficial high-intensity interval training, characterized by short intermittent bursts of vigorous exercise interspersed with periods of recovery provides a strong option for improving MQC in PD. According to one study, supervised high-intensity interval training appears to be feasible and acceptable for subjects with early- to mid-stage PD. Consistent with this finding, PD subjects were able to exercise at over 85% of their maximal heart rate across the 12-week intervention. Significant improvements in cardiorespiratory fitness were observed [154]. Low-volume, high-intensity interval training may even provide more benefits for PD patients.

### 2.11. Greater Metabolic Changes Induced by Exercise When the Exercise Occurs Early in the Active Period of the 24 h Circadian Cycle

A study using mice showed that exercise had different effects on metabolite levels of eight different tissues depending on whether the exercise occurred early in the active phase of the 24 h circadian cycle or 12 h later, early in the resting phase of the cycle [155]. A much more dramatic effect occurred when exercise was carried out early during the active phase of the circadian cycle following the fasted resting period that partially depleted liver glycogen levels. The levels of 197 metabolites were altered when exercise occurred during the active phase, compared to just 52 metabolite levels that were altered when exercise occurred during the resting phase. Plasma glucose and muscle glycogen levels decreased more and BHB levels increased more when exercise occurred early during the active phase, while levels of the stress hormone corticosterone and muscle lactate increased less. Hypothalamic serotonin and dopamine increased when exercise occurred during the active phase. Muscle and serum amino acids increased more during exercise in the active phase, which indicates the protein breakdown required for energy generation. Lipolysis of white adipose tissue was stimulated by exercise in the active phase, while lipolysis in the liver was stimulated by exercise during the resting phase.

Several protective compounds or “exerkines” were increased more during exercise early in the active phase, including serum AMP and beta-aminoisobutyric acid (BAIBA); muscle αkg, kynurenine, and GABA; liver BAIBA, kynurenine, and kynurenate; and hypothalamic BAIBA [155]. There was also increased muscle GSSG/GSH that occurred when exercise was carried out in the active phase. The more oxidized cellular environment following exercise in the active phase likely allows for more mitohormesis and ROS-induced Nrf2 activation several hours later, increasing NADPH levels and correcting the deficit. The most striking finding was the much greater global increase in 2-hydroxybutyrate (α-hydroxybutyrate) levels following active-phase exercise. In the same way that the pyruvate/lactate ratio is a marker of the cytoplasmic [NAD^+^]/[NADH] ratio due to the high activity of lactate dehydrogenase in most tissues [156], the 2-ketobutyrate/2-hydroxybutyrate ratio is also a global redox marker of the cytoplasmic [NAD^+^]/[NADH] ratio [157] due to 2-ketobutyrate and 2-hydroxybutyrate also serving as substrates for lactate dehydrogenase [158]. The more reduced [NAD^+^]/[NADH] ratio directly following exercise early in the active phase is likely an important signal to induce cytoprotective gene expression and metabolic changes leading to the restoration of the normal redox state.

The increased 2-hydroxybutyrate levels following active-phase exercise were shown to be due to increased flux through the transsulfuration pathway, where methionine is metabolized into 2-ketobutyrate, which can then be metabolized into 2-hydroxybutyrate. The transsulfuration pathway increases the generation of the cytoprotective gas hydrogen sulfide and generates increased levels of cysteine, the rate-limiting substrate required for glutathione synthesis [159]. When the cellular [NAD^+^]/[NADH] is in the normal oxidized state, the 2-ketobutyrate is transported into the mitochondrial matrix to form propionyl-CoA, which is largely metabolized into succinyl-CoA that enters the citric acid cycle. Conversely, when the cellular [NAD^+^]/[NADH] is reduced, cytoplasmic 2-ketobutyrate is instead reduced to 2-hydroxybutyrate. Increased transsulfuration pathway flux also occurs during anti-aging fasting or CR [160]. Increased transsulfuration pathway-mediated cysteine synthesis preserves cytoplasmic NADPH levels since following uptake from the bloodstream cellular cystine must be oxidized to cysteine using NADPH-derived reducing equivalents.

A study was performed where all experimental groups of mice were fed a calorie-restricted diet and had free access to a running wheel. One group with 24 h access to the food showed 11% extended longevity when compared to the control ad libitum-fed mice [161]. Time-restricted feeding was also employed by administering the same calorie-restricted diet only during a 12 h night-time (active period) or daytime (inactive period) timeframe. Administering the calorie-restricted diet only for the 12 h night-time (active) period extended the mean lifespan by 34%. Administering the calorie-restricted diet only during the 12 h daytime (inactive) period timeframe extended the lifespan by 19%. There were no differences in the weight of the mice among the three calorie-restricted groups. The study showed that fasting during the inactive portion of the 24 h cycle provided the most potent anti-inflammatory and anti-aging effects and provides a strong rationale for humans to fast at night and exercise during the day for optimal health benefits.

Rats fed a normal diet limited to ten hours in the daytime inactive period showed disrupted circadian rhythms in the rate of mitochondrial respiration in skeletal muscle. This mimics the metabolic dysfunction observed in human subjects with type-2 diabetes and in night-shift workers, who have a greatly increased risk of type-2 diabetes [61]. Therefore, fasting during the active period of the circadian cycle may result in an increased risk of metabolic disease if exercise is not performed or if calories are not restricted during the inactive feeding period.

### 2.12. Entrainment of the Circadian-Regulated Production of NAD^+^ in the Morning

Because NAD^+^ levels decline with aging in most tissues and this leads to decreased metabolic function, there is much clinical interest in the use of NAD^+^ precursors as compounds to increase cellular NAD^+^ levels to rejuvenate aged and diseased tissues [162]. Decreased cellular NAD^+^ levels also blunt beneficial signaling through sirtuin NAD^+^-dependent protein deacetylases [163]. In mice, the decreased NAD^+^ levels that occur with aging are largely due to increased levels and activity of the CD38 enzyme, which is most highly expressed in macrophages and hydrolyzes NAD^+^ as well as its direct precursor nicotinamide mononucleotide (NMN) both intracellularly and extracellularly [164]. The activity of NAMPT also declines slightly with aging [165]. Using NAD^+^ precursor supplementation to restore NAD^+^ levels has shown health benefits in several mouse models of disease that show substantially decreased NAD^+^ levels [166].

Cytoplasmic NAD^+^ is converted into NADP^+^ solely by the tetrameric NAD^+^ kinase (NADK) enzyme, which uses ATP to phosphorylate NAD^+^ [167], while mitochondrial NAD^+^ is phosphorylated solely by the dimeric NADK2 enzyme [168], which uses either ATP or polyphosphate as the phosphate donor [169]. NAD^+^ is transported into the mitochondrial matrix by the mitochondrial carrier family members SLC25A51 and SLC25A52 to gain access to mitochondrial NADK2. The cytoplasmic and mitochondrial [NADPH] to [NADP^+^] ratios are maintained at close to 100 to 1 [170]. Thus, small increases in NADP^+^ lead to large increases in NADPH. In support of this relationship NADK overexpression in cultured cells substantially increased NADPH levels with little effect on NADP^+^ levels [171].

Since oral intake of NAD^+^ precursors leads to their extensive metabolism by gut microbiota and first-pass hepatic metabolism [172], strategies to increase NAD^+^ levels in peripheral tissues are an active area of research [173]. Older methods aimed at replenishing cellular NAD^+^ levels include the oral consumption of vitamin B3 in the form of nicotinic acid or nicotinamide. The amino acid tryptophan can also be converted to NAD^+^, which occurs most readily in the liver. The liver releases nicotinamide as a major NAD^+^ precursor for most other tissues in the body [174]. The oral consumption of the more recently available NAD^+^ precursors, nicotinamide riboside and nicotinamide mononucleotide, has been successful in the treatment of many mouse models of disease, although, in many cases, this did not greatly increase the NAD^+^ levels in the peripheral tissues of young healthy mice [174]. A recent clinical trial has shown that nicotinamide riboside consumption can increase NAD^+^ levels in PD patient brains, which was correlated with a functional improvement in PD symptoms [175].

An alternative or complementary strategy to the oral supplementation of NAD^+^ precursors to raise the cellular NAD^+^ levels is to use fasting, exercise, and the body’s own natural circadian rhythms. During the early morning (4:00 AM) in humans, the heterodimeric transcription factor composed of the proteins BMAL1 (ARNTL) and CLOCK is most active in order to transcribe the NAMPT gene in skeletal muscle [176]. Therefore, during the early- to mid-morning the levels of NAD^+^ are likely at their highest point at least in skeletal muscle. SIRT1 consumes NAD^+^ in deacetylation reactions and specifically removes an acetyl group from a lysine residue in the BMAL1 transcription factor to activate the heterodimer of BMAL1 and CLOCK for transcription. The period (*PER1*, *PER2*, and *PER3*) genes are expressed in a circadian manner in the suprachiasmatic nucleus pacemaker. The PER proteins bind to CRY (CRY1 and CRY2) proteins to form a heterodimeric transcriptional repressor that further binds to and prevents the transcriptional activity of CLOCK-BMAL1 to restore the night-time pattern of gene expression. PER2 lysine acetylation increases PER2 function, and the acetyl group is removed by SIRT1, which inhibits protein function. The sum of these processes cause NAD^+^ levels to be entrained by the circadian transcription factors. Therefore, the activity of the heterodimeric transcription factor CLOCK-BMAL1, the transcription of the NAMPT gene, and NAD^+^ levels increase in unison [177]. In mice NAD^+^ levels are altered by up to 40% during different times of the 24-h cycle [178,179].

In contrast to peak human skeletal muscle NAD^+^ levels that occur in the morning, the circadian oscillations in human mitochondrial respiration peak at roughly 11:00 PM, with the trough occurring at 1:00 PM [180]. The reason for why these different metabolic oscillations are not in phase is not well understood. In studies of humans performing roughly 14 h fasts, it was shown that fasting from dinner until breakfast led to more lipid oxidation than fasting from bedtime until lunch [181]. These results may be due to increased rates of fatty acid beta-oxidation during fasting and the circadian increase in ETC function in the evening. These events lead to higher rates of fatty acid beta-oxidation during evening fasts compared to those that occur during morning fasts, when diurnal ETC activity is not at its peak.

Unlike humans, mice are nocturnal. NADK is also expressed in a highly circadian fashion and is expressed most highly in the afternoon in the mouse liver, before the mice become active in the evening [182]. Circadian NADK expression mirrors the circadian expression of HMGCR, the rate-limiting enzyme of the cholesterol biosynthesis pathway, presumably due to the large amount of NADPH oxidized by this pathway. If circadian gene expression in human neurons follows that in skeletal muscle and liver, there should be an increase in both NAD^+^ and NADPH levels in neurons in the morning.

### 2.13. Circadian Regulation of Mitophagy and Mitochondrial Dynamics May Play a Role in PD

There is much evidence indicating altered circadian physiology in PD patients [183] and pre-clinical models of PD [184]. For example, 65% to 95% of PD patients exhibit sleep impairments [185]. This may partly be due to the regulation of the circadian clock by dopamine [186,187], which is deficient in PD subjects. Dopamine regulates the expression of PER2 [188], as well as the transcriptional activity of the CLOCK/BMAL1 heterodimer [189,190]. The tyrosine hydroxylase gene, as well as dopamine receptor genes, are expressed in a circadian manner [191]. The decreased dopamine and melatonin levels and increased cortisol levels in PD patients are associated with decreased BMAL1 expression [192,193]. Long term L-DOPA therapy [194] or increased neuroinflammation [195] were shown to accelerate the circadian dysfunction in PD rat models. The circadian regulators BMAL1 [196] and REV-ERBα [197] have been shown to preserve motor function and reduce neuroinflammation in mouse models of PD. The role of alterations in circadian gene expression in neuroinflammation and the pathogenesis of PD has recently been reviewed [183,198].

Not only does mitochondrial ETC complex activity follow circadian rhythms [199], but mitophagy rates and mitochondrial dynamics also follow a diurnal pattern [200]. In the liver, BMAL1 induces the expression of PINK1, the mitochondrial fission protein FIS1, and the mitophagy receptor BNIP3. The expression of the mitochondrial fission protein DRP1 also fluctuates in a diurnal pattern [201]. The loss of the CLOCK protein disrupts the expression of the autophagy proteins ATG7, RAB7a, TFEB, and SQSTM1 [202]. Conversely, *Parkin* mutations in PD patients also disrupt circadian gene expression in iPSC-derived neurons [202]. HDAC3 negatively regulates the circadian clock and mitophagy by activating Rev-erbα, a negative regulator of BMAL1 [181], and possibly by inhibiting PGC-1α expression [203]. Consistent with these results, murine embryonic fibroblasts (MEFs) from Rev-erbα transgenic mice exhibit increased mitochondrial mass and respiration due to a 4-fold decrease in mitophagy [204]. Streptozotocin-induced diabetes in rats upregulated HDAC3 to blunt circadian gene expression and mitophagy [204]. Fasting and ketone ester-induced increases in BHB levels may therefore prevent the decreased mitophagy in PD cells by increasing NADPH levels to inhibit HDAC3 [9]. In tissues where BHB does not increase NADPH levels, longer-term human fasting (>48 h) to induce millimolar levels of BHB may also inhibit HDAC3 activity directly [205].

## 3. Dopaminergic Neurons Are Vulnerable to Dysfunctional MQC

The discovery that the PD-causing toxin MPTP was converted to MPP^+^ by the enzyme monoamine oxidase-B (MAO-B) to cause dopaminergic neuron loss and PD symptoms led to a quest to find the mechanism underlying neuronal toxicity. MPTP was found to be taken up by astrocytes and serotonergic neurons that express MAO-B and then converted into MPP^+^, which was released into the extracellular space. MPP^+^ was then found to be taken up by dopamine transporters expressed in dopaminergic neurons [206]. From the cytoplasm, MPP^+^ is concentrated 40-fold in the mitochondrial matrix [207], resulting in the inhibition of ETC complex I. Therefore, the most efficient mitochondria with the highest membrane potential are the ones that accumulate the most MPP^+^ resulting in the most ETC inhibition [207].

Does the inhibition of complex I by MPP^+^, rotenone, or alpha-synuclein [208] depolarize the mitochondrial inner membrane enough to stimulate mitophagy when complex II is still active for electron transport? The answer may depend upon the cell type studied. However, even if mitochondrial depolarization does not occur immediately, it likely eventually occurs because of the increased O_2_^•−^ generation that results from complex I inhibition, leading to increased ONOO^−^ and H_2_O_2_ formation, which open the mPTP to completely depolarize the mitochondrion [209]. In either case, the data strongly suggest that interfering with MQC leads to PD. Therefore, PD treatments should be directed towards increasing mitochondrial resilience by improving the redox state.

**Figure 4:**
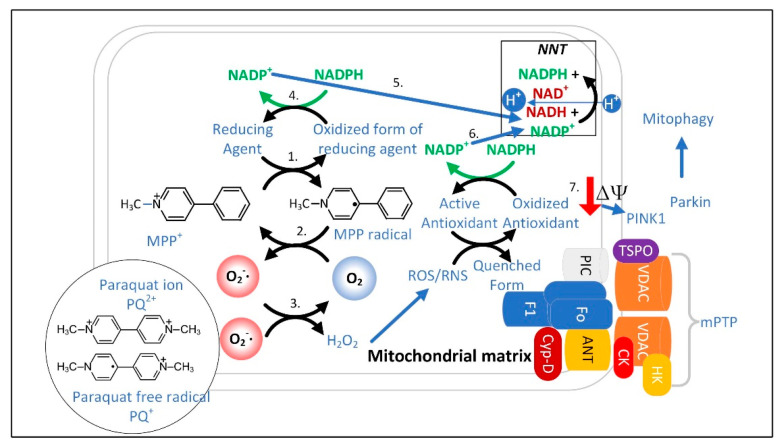
MPP^+^ redox cycling occurs in mitochondria with a high membrane potential and leads to depolarization and mitophagy. 1. MPP^+^ is reduced to MPP^•^. 2. O_2_ oxidizes MPP^•^ back to MPP^+^ and generates O_2_^•−^. 3. O_2_^•−^ is detoxified by SOD2. 4. NADPH donates electrons to reduce the oxidized form of the reducing agent. 5. Mitochondrial NADPH levels decrease and NNT activity restores NADPH levels. 6. The formation of other ROS further depletes NADPH. Increased NNT activity and mPTP opening depolarizes the inner mitochondrial membrane, leading to the translocation of PINK1 from the inner to the outer mitochondrial membrane. 7. Outer membrane PINK1 recruits Parkin leading to mitophagy. The PD-causing herbicide paraquat undergoes redox cycling between the PQ^2+^ ion and the PQ^•+^ free radical to induce a similar sequence of events. Abbreviations: translocator protein of 18 kDa (TSPO), voltage-dependent anion channel (VDAC), hexokinase (HK), adenine nucleotide transporter (ANT), phosphate carrier protein (PIC), cyclophilin-D (Cyp-D), subcomplexes of ATP synthase (F1), (F0), and creatine kinase (CK).

MPP^+^ concentrated in the mitochondrial matrix undergoes redox cycling [210], a futile oxidation-reduction cycle that generates O_2_^•−^ (Figure 4) [211]. The redox potential for the conversion of MPP^+^ to MPP^•^ radical is approximately −1.0 V. The mitochondrial or cytoplasmic [NADP^+^]/[NADPH] has a redox potential of only −0.42 V [170], and therefore, NADPH cannot reduce MPP^+^. Two-electron reduction pathways have a higher redox potential of approximately −0.84 V. Thus, two-electron reducing agents present in the mitochondrial matrix were sought, and two candidates, ALDH2 (aldehyde dehydrogenase H2) and DLD (dihydrolipoamide dehydrogenase), that could reduce MPP^+^ were identified [210,212].

### 3.1. The Superoxide Sentinel Hypothesis of MQC

**Figure 5:**
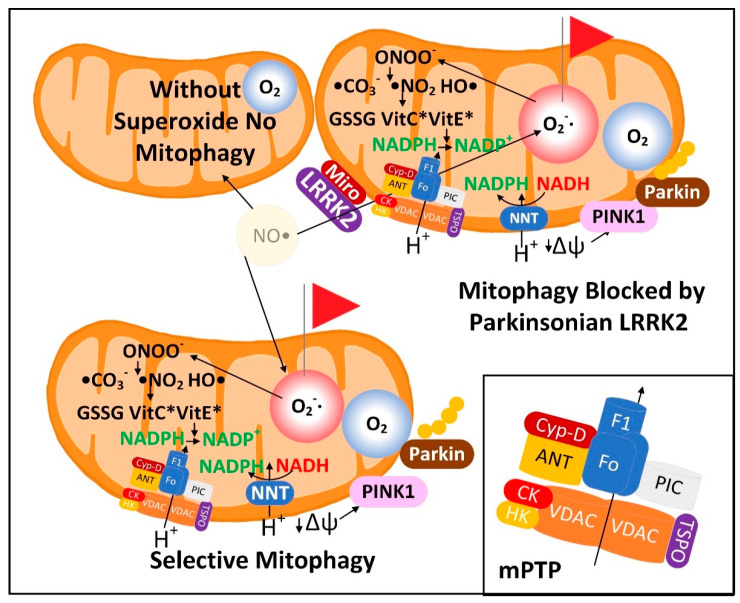
Mitochondrial matrix space O_2_^•−^ may function as a sentinel for mitophagy by reacting with ^•^NO to form ONOO^−^ that dissipates the mitochondrial membrane potential, leading to PINK1- and Parkin-mediated mitophagy. PD-inducing mutations in LRRK2 can block mitophagy.

When mitochondria are old or damaged the ETC is less efficient and more O_2_^•−^ is formed. This common feature of dysfunctional mitochondria makes O_2_^•−^ an ideal sentinel for marking the mitochondria for mitophagy. It also suggests that dysfunctional MQC is associated with a heavier load of other ROS/RNS, such as H_2_O_2_, which is synthesized from O_2_^•−^ by mitochondrial SOD2. H_2_O_2_ could also potentially be used to identify damaged mitochondria [213]. Although both O_2_^•−^ and H_2_O_2_ can stimulate autophagy, SOD2 overexpression, which increases H_2_O_2_ levels, decreased autophagy, while SOD2 knockdown, which increases O_2_^•−^ levels, increased autophagy [31]. Other studies have also shown that O_2_^•−^ is a much more potent stimulator of mitophagy than H_2_O_2_ [214,215]. This is consistent with a superoxide sentinel hypothesis that proposes that high matrix space levels of O_2_^•−^ mark the organelle for mitophagy (Figure 5). O_2_^•−^ is negatively charged and thus impermeable to phospholipid bilayers. Therefore, most of the O_2_^•−^ that is released into the matrix space remains there until it is detoxified or causes damage. However, there are inner mitochondrial membrane anion channels that function in mitochondrial volume regulation that transport O_2_^•−^ out of the matrix space when they are open [216]. However, due to the short half-life of O_2_^•−^, little is likely exported to the cytoplasm. However, the mitochondrial ETC also releases some O_2_^•−^ into the intermembrane space, where SOD1 is present to detoxify most of it to H_2_O_2_. 

H_2_O_2_ is relatively polar and cannot readily diffuse through the mitochondrial inner membrane. So, it is transported out of the mitochondrial matrix space through aquaporin channels in the inner membrane [217,218]. The ability of H_2_O_2_ to be released into the cytoplasm allows it also to serve as a signal of the mitochondrial function. Mitochondrial H_2_O_2_ is detoxified in the matrix space into water by the enzymes peroxideroxin 3 (PRDX3) and peroxiredoxin 5 (PRDX5) as well as by glutathione peroxidase using glutathione (GSH). GSH is transported into the mitochondrial matrix by the SLC25A39 and SLC25A40 carriers [219]. Mutations in SLC25A39 have been linked to late-onset PD [220]. Oxidized mitochondrial PRDX3 and PRDX5 are reduced by thioredoxin 2 (TXN2). Oxidized glutathione disulfide (GSSG) and TXN2 are reduced back to their active forms by glutathione reductase (GSR) and thioredoxin reductase 2 (TXNRD2), respectively, oxidizing NADPH into NADP^+^. By mass action, this decrease in the matrix space NADPH activates mitochondrial NNT to synthesize NADPH. Under normal conditions, in the absence of redox cycling, proton transport by NNT only slightly depolarizes the inner mitochondrial membrane. NNT is expressed at moderately low levels in neurons, most notably in neurons that synthesize serotonin and neuronal nitric oxide synthase (nNOS). Its expression in astrocytes and microglia is very low [221].

There are several mitochondrial targets through which ^•^NO or ONOO^−^ bind to and additively disable mitochondrial function to increase flux through the NNT and thus to significantly depolarize the inner mitochondrial membrane. ^•^NO is a free radical that inhibits mitochondrial ETC complex IV by competing for the binuclear binding site for O_2_ [222]. This complex IV inhibition results in the formation of O_2_^•−^ from complex III [223,224]. ONOO^−^ binds and inhibits SOD2 to increase matrix space O_2_^•−^ levels [225]. Locally high concentrations of CO_2_ synthesized by the PDC, isocitrate dehydrogenase, and the αkg dehydrogenase complex react with ONOO^−^ to form CO_3_^−•^ and NO_2_^•^. ONOO^−^ also undergoes a proton-catalyzed homolysis reaction producing hydroxyl radical (^•^OH) and NO_2_^•^ radical [226]. These mechanisms may lead to substantially increased mitochondrial ROS levels and decreased NADPH levels, thus greatly increasing NNT activity to facilitate mitochondrial depolarization. However, ONOO^−^ has also been shown to bind to and inhibit NNT, which suggests that the mPTP is a more likely candidate for mediating the ROS-induced mitochondrial membrane depolarization to signal for mitophagy [227]. Decreased mitochondrial matrix NADPH levels also facilitates the opening of the mPTP [228] to depolarize the mitochondrion [229]. The genome of C57BL/6J mice contains a mutation inactivating the NNT, but the nigrostriatal system is largely unaffected [230], likely due to the compensatory synthesis of NADPH by other cellular enzymes [231]. No defects in neural mitophagy have been ascribed to NNT deficiency either, even though mitochondrial dynamics and turnover are well known to be affected by the cellular redox state [232].

**Figure 6:**
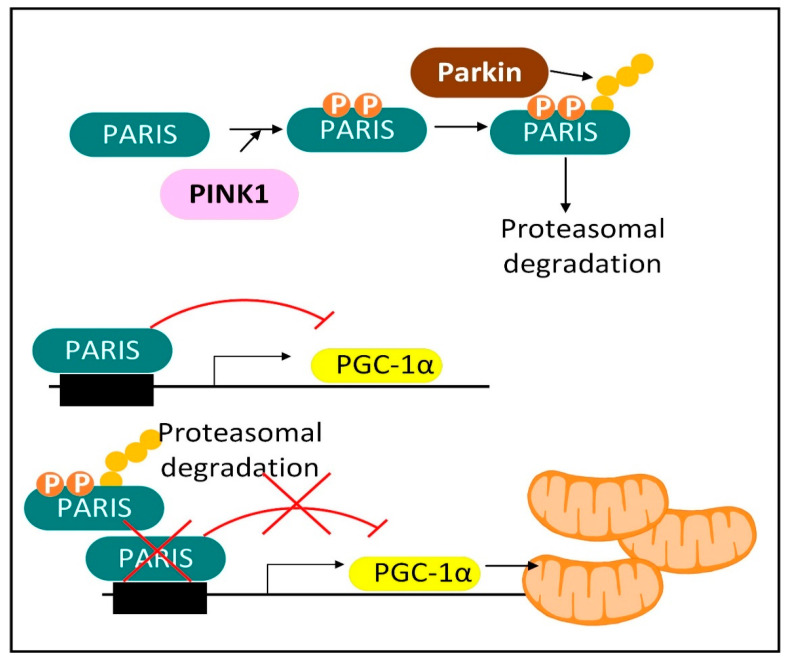
Mitochondrial biogenesis triggered by PINK1 and Parkin. PINK1 and Parkin stimulate mitochondrial biogenesis by causing the phosphorylation, ubiquitination, and proteasomal degradation of PARIS, an inhibitor of PGC-1α expression.

Mitochondrial depolarization is known to activate PINK1 protein kinase by altering its mitochondrial localization. Mutated *PINK1* is one of the autosomal recessive genes that, when mutated, causes PD. PINK1 interacts with Parkin to initiate mitophagy and mitochondrial biogenesis (Figure 6). Mitochondrial biogenesis is stimulated by PINK1-induced phosphorylation and Parkin-induced ubiquitination and proteasomal degradation of Parkin-interacting substrate (PARIS). PARIS is a negative regulator of the PGC-1α promoter. Therefore, PINK1 and Parkin-induced PARIS degradation functions to increase the expression of PGC-1α leading to mitochondrial biogenesis. *Parkin* knockout also decreased SIRT1 levels. Thus, increased SIRT1 levels may also play a role in PINK1–Parkin-mediated mitochondrial biogenesis. *Parkin* is an autosomal recessive gene that when mutated causes PD. There is also a PINK1- and Parkin-independent mitophagy pathway [233]. Once a neuron possesses a MQC deficit, there are several downstream events that perpetuate and exacerbate neuronal dysfunction. These events may be sufficient to explain the continued physiological decline and progression of PD over many years.

### 3.2. Neurons with Low-Quality Damaged Mitochondria Likely Show Decreased Proteolysis of α-Synuclein

Postmitotic cells such as neurons cannot dilute cytoplasmic contents through cell division, and so high concentrations of oxidized and aggregated proteins such as α-synuclein can potentially accumulate. Therefore, postmitotic cells rely on efficient proteolytic systems to prevent protein accumulation. The α-synuclein protein can undergo several post-translational modifications that favor aggregation and lead to toxicity [234]. The cell has two major proteolytic systems for clearing damaged or aggregated cytoplasmic proteins such as α-synuclein [234], and they both require sufficient ATP. In the ubiquitin-proteasome system, ubiquitin is covalently attached to α-synuclein or another protein that exposes a hydrophobic core on the surface of the protein. Both the post-translational modification attaching ubiquitin to the protein to be degraded and the proteolysis by the proteasome require ATP [235,236]. Autophagy is the other major proteolytic system used to degrade α-synuclein and other cytoplasmic proteins and organelles. Autophagy results in complete lysosomal proteolysis yielding individual amino acids. The various types of autophagy, including macroautophagy, microautophagy, and chaperone-mediated autophagy, all require ATP [237]. 

Given the diminished glycolysis [238] and mitochondrial ETC activity that occurs in PD dopaminergic neurons and in other neurodegenerative disorders characterized by dysfunctional MQC, one important question is whether glucose and fatty acids are still capable of supplying the ATP required for the proteolytic processes that remove damaged and misfolded proteins such as α-synuclein. If glucose and fatty acids are not sufficient, can levels of the ketone body BHB be raised high enough to produce sufficient ATP by oxidative phosphorylation? Unfortunately, all mitochondrial fuels are probably oxidized more slowly in PD neurons that possess ETC dysfunction. This dysfunction results in a decreased mitochondrial matrix [NAD^+^]/[NADH] ratio that slows citric acid cycle activity and inhibits other fuel-specific mitochondrial NAD^+^-dependent catabolic enzymes, such as PDC, BHB dehydrogenase (BDH1), and 3-hydroxyacyl-CoA dehydrogenase.

The use of glucose as a fuel source for ATP synthesis faces some initial hurdles not faced by ketone bodies. For example, the cellular uptake of glucose for glycolysis is dependent on glucose transporter localization to the plasma membrane, which is reliant on the release of insulin by pancreatic islets and the successful binding of insulin to plasma membrane insulin receptors, as well as the activation of the AKT signaling pathway [239]. Cytoplasmic glucose metabolism also has an upfront cost of one ATP molecule when the glucose molecule enters the cell and is phosphorylated by hexokinase. The glycolysis end-product, pyruvate, is transported into the mitochondrial matrix and metabolized by the highly regulated PDC, which is frequently phosphorylated and inhibited by one of four pyruvate dehydrogenase kinase (PDK) isoforms in the brain of individuals affected by neurodegenerative disorders. Although cytoplasmic pyruvate can then be reduced to lactate, this entails a large loss of ATP synthesis when compared to the amount that would have been generated by oxidative phosphorylation. Enhancing glycolysis has been shown to improve PD symptoms in humans and in animal models of PD [240].

The use of fatty acids as an energy source for oxidative phosphorylation also faces some initial hurdles. The BBB limits the transportation of some long-chain and very long-chain fatty acids to the central nervous system, but not the short-chain, medium-chain, or essential long-chain fatty acids [241]. Therefore, the neural cells must synthesize some of their own fatty acids and this requires sufficiently high levels of NADPH. Extracellular fatty acids are transported by apolipoprotein carriers that dock at the plasma membrane of neural cells and release the fatty acids for cellular uptake. Neurons are known to express low levels of mitochondrial fatty acid synthesis and beta-oxidation enzymes [242]. Thus, some fatty acids are synthesized in astrocytes and then delivered to neurons [243], while neuronal oxidatively damaged fatty acids are transported in the reverse direction to astrocytes for fatty acid oxidation [244]. Long-chain fatty acids have an upfront cost of one ATP to enter the mitochondrial matrix through the malonyl-CoA-inhibited carnitine palmitoyl transferase (CPT1) system. 

Unlike glucose or fatty acids, the ketone body BHB is transported into neural cells through monocarboxylate transporters, where there is no initial ATP requirement for metabolism. Secondly, there is no highly regulated mitochondrial gatekeeper, such as PDC or CPT1, for ketone body catabolism. Unlike glucose, which is stored as glycogen, and fatty acids, which are stored as triacylglycerides, BHB is inefficiently stored in eukaryotic cells, where only a very small amount of short-chain (<30 monomers) poly-β-hydroxybutyrate polymers accumulate in mitochondrial membranes [245]. Thus, when BHB is taken up by a cell, the vast majority is quickly transported into the mitochondrial matrix and used as a fuel to synthesize ATP. In tissues that synthesize ketone bodies, such as the liver and kidneys, the level of BHB increases with fasting, leading to its reaction with coenzyme A to form beta-hydroxybutyryl-CoA, which is used as a substrate for protein lysine beta-hydroxybutyrylation [246]. Protein lysine beta-hydroxybutyrylation also occurs during fasting in the small intestine [247]. The enhanced ATP levels that occur when PD neural cells oxidize ketone bodies could contribute to increased activity of the proteolytic machinery to degrade α-synuclein and misfolded proteins. However, ketone bodies are only present in sufficient quantities for oxidation during fasting, during a ketogenic diet, or following the consumption of exogenous ketone bodies or their precursors. 

### 3.3. Limited Clearance of ROS/RNS Leads to the Oxidation and Depletion of Cardiolipin and Plasmalogens

Another outcome of poor MQC is the increased oxidation of polyunsaturated fatty acids by ROS to form isoprostanes, malondialdehyde, or 4-hydroxynonenal (4-HNE), which inhibit the function of proteins. For example, IDH1 is susceptible to covalent modification and inhibition by 4-HNE [248]. 4-HNE is produced by ^•^OH attacking polyunsaturated fatty acids. Limiting dietary omega-6 polyunsaturated fatty acids, such as those that are present at high levels in vegetable oils (excluding olive oil), may be beneficial in decreasing 4-HNE synthesis. Consistent with this hypothesis, high levels of the omega-6 fatty acids dihomo-gamma-linolenic acid (DGLA) [249] and arachidonic acid (AA) [250] have been shown to cause ferroptosis. PD neural cells are characterized by lipidopathy, particularly of sphingolipids. PD neurons and microglia are characterized by increased levels of fatty acids and lipid droplets, while PD astrocytes show decreased levels of these lipids [251].

Poor MQC also leads to the oxidation and depletion of mitochondrial cardiolipin (a diphosphatidylglyceride lipid with two phosphate groups and four acyl chains) and plasmalogens (vinyl-ether phospholipids enriched with AA and docosahexaenoic acid (DHA)). Cardiolipin constitutes roughly 20% of the inner mitochondrial membrane phospholipids and is critical for the mitochondrial inner membrane to maintain a membrane potential, as each cardiolipin molecule can bind a proton. When oxidatively damaged cardiolipin binds cytochrome c it activates a latent peroxidase [252] and plasmalogenase [253] activity, which can contribute to cell death. *PLA2G6*, group VIA calcium-independent phospholipase A2-beta (iPLA2-β), is a gene that when mutated causes PD, and the gene product has a partial mitochondrial localization. iPLA2-β [254], as well as the related mitochondrial and peroxisomal-localized iPLA2-ɣ [255], can hydrolyze fatty acids from cardiolipin. The depletion of cardiolipin can cause a direct inhibition of mitophagy, as the redistribution of cardiolipin from the inner mitochondrial membrane to the outer mitochondrial membrane is an important step in mitochondrial fission and mitophagy. The Drp1 protein that is recruited to the mitochondria to mediate mitochondrial fission [256] and the LC3/Atg8 protein involved in autophagosome formation [257] bind cardiolipin as important steps in MQC. Cardiolipin is also required for the activity of the NLRP3 inflammasome [258]. So, cells may have evolved to decrease cardiolipin levels in times of oxidative stress to reduce inflammation.

Plasmalogens are important structural components of membranes in the brain, where they comprise 20% of the total phospholipids. They play an especially important role as a constituent of the myelin sheath surrounding the axons, where ethanolamine plasmalogens comprise 70% of the phospholipids. The ethanolamine plasmalogens are required for neurotransmitters to be packaged into stable vesicles docked near the plasma membrane. Plasmalogens support the fusion of the neurotransmitter vesicles with the presynaptic plasma membrane, allowing evagination and release into the synapse. The highly unsaturated fatty acids in cardiolipin make it susceptible to oxidation, while the ether bond in the plasmalogens makes them susceptible to oxidation. Mitochondrial depletion of these phospholipids is an early event in Alzheimer’s disease (AD) model mice [259]. In Barth syndrome plasmalogen loss occurs because of decreased remodeling of fatty acids in cardiolipin due to tafazzin deficiency [260]. Thus, the stability of cardiolipin and plasmalogens appears to be indirectly linked.

The plasmalogens present in foods are degraded in the acidic environment of the stomach, and therefore, cells must synthesize plasmalogens from precursors. Plasmalogen levels decline with aging and decline further in the brain when affected by neurodegenerative disorders such as PD and AD [261,262,263]. Plasmalogen synthesis is initiated in peroxisomes and then continues in the ER [264]. Peroxisomal fatty acid oxidation leads to high levels of H_2_O_2_ generation [265]. Peroxisomal dysfunction, in part due to oxidative damage by ROS/RNS, may be responsible for the decreased levels of plasmalogens that occur in aged and diseased tissues [266]. Plasmalogen levels can be increased by taking plasmalogen precursors as supplements [261]. While some studies demonstrate sufficient plasmalogen precursor BBB permeability [262], others suggest that these supplements are not sufficiently permeable through the BBB and that the consumption of other supplements such as DHA [267], ethanolamine, or myoinositol [268], may be consumed instead to increase brain plasmalogen levels. The aging-related oxidation and loss of plasmalogens [163] is likely delayed by fasting or CR that increase ketone body levels that boost the activity of Nrf2, which increases antioxidant gene expression and NADPH levels to provide reducing power to cellular antioxidants [82].

Lysosomal dysfunction plays a prominent role in PD, as roughly half of the inherited cases of PD occur due to mutations in the genes involved in lysosomal storage disorders. Heterozygous mutations in GBA, the gene encoding the enzyme glucocerebrosidase, increases the risk of PD while homozygous mutations in GBA lead to Gaucher disease. Lysosomal storage disorders are characterized by the lysosomal accumulation of the unique glycerophospholid bis(monoacylglycerol) phosphate (BMP), also called lysobisphosphatidic acid (LBPA), which may deplete phosphatidylglycerol levels, decreasing the rate of synthesis of mitochondrial cardiolipin, which is required for ETC function. Lysosomal dysfunction also interferes with the normal subcellular distribution of ceramide and cholesterol to mitochondria and other organelles, altering mitochondrial function [269] and dynamics [270] and contributing to cellular dysfunction in PD.

### 3.4. NADPH May Decrease in PD Neurons and Glia Limiting the Synthesis of Serotonin, Melatonin, Epinephrine, Norepinephrine, and •NO

**Figure 7:**
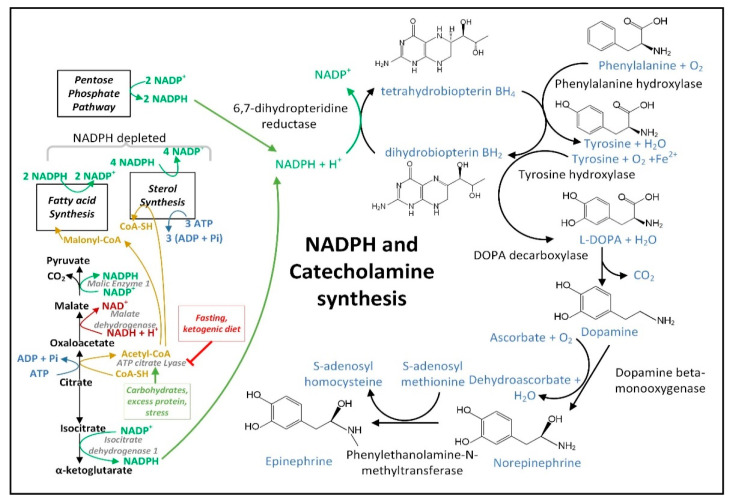
NADPH and BH_4_ are required for the synthesis of dopamine, norepinephrine, and epinephrine. Fasting likely diverts more cytoplasmic citrate to isocitrate, which is metabolized by IDH1 to increase cytoplasmic NADPH levels, while carbohydrate consumption activates ATP-citrate lyase metabolism of cytoplasmic citrate to increase fatty acid synthesis, which depletes cytoplasmic NADPH.

The increase in the cellular [NADP^+^]/[NADPH] ratio in PD neural cells resulting from dysfunctional MQC and increased O_2_^•−^ generation may decrease astrocyte NADPH-dependent fatty acid synthesis, thus decreasing the quantity of neutral lipids [271] and may also decrease the NADPH-dependent neuronal synthesis of many neurotransmitters. Due to a decline in NADPH, the active coenzyme tetrahydrobiopterin (BH_4_) becomes oxidized to the inactive form biopterin (BH_2_), and thus becomes unavailable in the quantities required for efficient synthesis of dopamine [229], epinephrine, and norepinephrine (Figure 7), as well as serotonin, melatonin, and ^•^NO. Decreased mitochondrial NADPH can also limit the synthesis of proline required for protein synthesis [272].

### 3.5. Insulin Released after Feeding Increases Cytoplasmic NADPH Oxidation by Stimulating Fatty Acid Synthesis

The decreased hepatic cytoplasmic [NADP^+^]/[NADPH] ratio that occurs during fasting may require glucagon [273] and may not occur after carbohydrate consumption in part due to insulin signaling. When insulin binds its receptor on the plasma membrane, a signaling cascade is initiated activating protein kinase AKT that phosphorylates ATP-citrate lyase (ACLY) serine 454 to activate the enzyme [274]. Thus, the citrate that is exported from the mitochondrial matrix is diverted away from the citrate–αkg shuttle that uses IDH1 for NADPH synthesis to boost cytoplasmic NADPH levels, and instead it is routed to the citrate–malate or citrate–pyruvate shuttles that use ACLY for the synthesis of acetyl-CoA and oxaloacetate. Acetyl-CoA is largely used for NADPH-depleting fatty acid synthesis. The malate dehydrogenase 1 (MDH1) enzyme converts the generated oxaloacetate into malate, which can either be transported back into the mitochondrial matrix for the citrate–malate shuttle or converted to pyruvate by cytoplasmic ME1 with the concurrent reduction of NADP^+^ to NADPH as part of the citrate–pyruvate shuttle. The pyruvate is then transported back into the mitochondrial matrix. 

During fatty acid synthesis, for an acetyl-CoA molecule to add two carbons to a growing fatty acyl chain, two NADPH molecules must be oxidized. This leads to a net decrease in the cytoplasmic NADPH levels when PPP activity and serine catabolism are not considered. To make up for this deficit in most tissues, each glucose molecule metabolized by the oxidative phase of the PPP reduces two NADP^+^ molecules to NADPH. Cytoplasmic citrate is also an allosteric activator of acetyl-CoA carboxylase 1 (ACC1) [275], which further stimulates ACLY activity by relieving the product inhibition by acetyl-CoA. To aid in the conversion of glucose to fatty acids and prevent decreased levels of NADPH, AKT also phosphorylates and activates NADK to increase NADP(H) levels [276]. NADK activity can also be increased by binding to the PPP enzyme G6PD. Thus, increased expression of G6PD increases NADK activity to further increase NADPH levels [277].

Fasting-induced ketosis yields low insulin and high glucagon levels stimulating the hepatic synthesis and peripheral catabolism of BHB in mitochondria to increase the levels of mitochondrial acetyl-CoA and citrate resulting in the release of citrate into the cytoplasm. Therefore, in dopaminergic neurons in the absence of insulin signaling the increased levels of cytoplasmic citrate are more readily metabolized by aconitase (ACO1) yielding isocitrate than by ACLY. The isocitrate is then converted into α-ketoglutarate by IDH1 leading to a reduced cytoplasmic NADP^+^/NADPH couple as no acetyl-CoA is produced for NADPH-depleting fatty acid synthesis. The increased level of cytoplasmic NADPH can therefore be used to maintain BH_4_ in the reduced form, which is required for the conversion of phenylalanine to tyrosine and for the conversion of tyrosine to L-DOPA, the direct precursor of dopamine. 

During times of fasting, when glucagon is high and insulin is low, glucagon binds to its cellular receptor to activate AMP kinase (AMPK) and protein kinase A (PKA) in many tissues including brain leading to the phosphorylation and inhibition of ACC1 [278]. This decreases malonyl-CoA production to decrease fatty acid synthesis and conserve NADPH. In addition to glucagon, ghrelin is another hormone released during the fasted state that mediates some of the neuroprotective effects of CR on dopaminergic neurons in PD model mice [279]. Ghrelin activates AMPK to induce mitophagy. Consistent with this, an AMPK agonist has been shown to improve motor functions in mice [280]. Intermittent AMPK activation was shown to be neuroprotective, while sustained AMPK activation was shown to impair neuronal plasticity, highlighting the important role of recovery in between stress-inducing events [281].

Carbohydrate consumption, which stimulates PPP-mediated NADPH synthesis leads to insulin release and increased energy production inhibiting AMPK activation and stimulating ACLY activity and fatty acid synthesis. Excess protein consumption can have a similar effect due to the stimulation of hepatic gluconeogenesis, which increases glucose levels. The detrimental effects of a high-protein meal on PD symptoms may be partly due to the gluconeogenic conversion of amino acids into glucose, the conversion of glucose into acetyl-CoA, and then the neuronal oxidation of NADPH by de novo fatty acid synthesis, which decreases the NADPH and BH_4_ availability for dopamine synthesis.

When ketone ester was first synthesized and tested in experimental models and humans, there was a naïve optimism that its exogenous consumption when taken together with a meal would nearly completely mimic the beneficial effects of intermittent fasting or CR in delaying aging and disease. Data obtained over many years have shown that although ketone ester consumption together with food can slightly delay the symptoms in several rodent disease models, its effects are much less dramatic than those of intermittent fasting or CR. Unpublished case reports on the human consumption of ketone ester suggest that ketone ester was able to improve the subject’s sense of well-being when it was consumed in the morning after an overnight fast. Therefore, many of the beneficial effects of BHB may depend on the fasted state, a time when insulin levels are low, and glucagon and ghrelin levels are high. When ketone bodies are present in tissues together with hormones that are normally present in the fasted state, a natural synergy likely occurs, and the benefits of longer-term fasting may be obtained from shorter-term fasts. A popular ketone ester is R-3-hydroxybutyl-(R)-3-hydroxybutyrate. It is hydrolyzed in the gut to BHB and (R)-1,3-butanediol. Surprisingly, (R)-1,3-butanediol was shown to protect against aging-associated vasculature dysfunction independently of its conversion to BHB in the liver [282].

### 3.6. Experiments Are Needed to Determine the (Free) Cytoplasmic [NADP^+^]/[NADPH] Ratio in Neural Cells from Aged and PD Model Mice and How These Are Altered by Fasting

When measuring total levels of NADP^+^ and NADPH, it was surprisingly shown that the mouse brain NADP^+^/NADPH ratio decreased with aging, mostly due to increases in NADPH levels [283]. The brain NADH level also increased with aging. In liver and brown adipose tissue NADPH levels declined with aging as would be expected for a condition characterized by increased oxidative stress. However, aging-induced NADPH loss was not observed in most tissues. Due to its known health benefits, fasting might be expected to universally reduce the total NADP^+^/NADPH ratio to combat oxidative stress. But in rat heart and skeletal muscle fasting led to an oxidation of this coenzyme couple. So redox changes are tissue specific. Measurements of total pyridine nucleotide ratios are also misleading and do not reflect the relevant (free) cytoplasmic [NADP^+^]/[NADPH] ratio, as much more NADPH than NADP^+^ is bound to protein and unavailable for enzyme function. Measurements of the pyruvate/malate ratio or αkg/isocitrate ratio have been commonly used to estimate the (free) cytoplasmic [NADP^+^]/[NADPH] in liver [82]. Results from a first attempt at using these metabolite ratios from whole brain to estimate cytoplasmic [NADP^+^]/[NADPH] yielded a promising average value across neural cell types [284].

The NAD(P)H level has been shown to decrease from middle to old age in isolated neurons [285,286,287]. However, it was not determined whether this decline was due to decreased levels of NADH or NADPH, since the cellular levels of both are of the same magnitude [36,288]. Since NADH and NADPH increased with aging in whole brain, but the NAD(P)H level decreased with aging in isolated neurons, the increase in brain NADH and NADPH with aging must be due to increased levels in glia. Increased NADPH levels in aged astrocytes and microglia may contribute to increased ROS production by NADPH oxidase enzymes found at high levels in these cell types. Since glial cells can have much higher PPP activity than neurons, fasting-induced decreases in glucose levels and PPP activity could potentially decrease NADPH levels in glial cells to decrease NADPH oxidase activity and ROS levels to stabilize the redox state. Pathologically high NADPH levels in astrocytes may also keep DJ-1 in the inactive sulfhydryl form to prevent Nrf2 activation as mentioned earlier to feedback inhibit NADPH synthesis.

### 3.7. Dysfunctional MQC Leads to Loss of Circadian Entrainment of NAD^+^ Synthesis

Dysfunctional MQC in PD decreases oxidative phosphorylation and shifts more of the burden of ATP synthesis to glycolysis. Increased glycolysis stimulates the rate of conversion of NAD^+^ to NADH by the GAPDH enzyme decreasing the cytoplasmic NAD^+^/NADH ratio and SIRT1 activity if the combined NADH oxidizing activities of mitochondrial ETC complex I and cytoplasmic lactate dehydrogenase do not increase in parallel. The ETC complex I dysfunction in PD [289] decreases the mitochondrial NAD^+^/NADH ratio, but this reduced mitochondrial NAD^+^/NADH ratio is not efficiently transferred to the cytoplasm due to the irreversibility of the malate–aspartate shuttle due to the coupling of the mitochondrial glutamate import (and aspartate export) to the proton-motive force during SLC25A12 and SLC25A13 carrier function [290]. This irreversibility maintains the roughly 100-fold higher [NAD^+^]/[NADH] ratio in the cytoplasm as compared to the mitochondrial matrix. The [NAD^+^]/[NADH] ratio is roughly 700 in the cytoplasm and is roughly 7 in the mitochondrial matrix [156].

As a result of brain aging, PD, and the accompanying dysfunctional MQC, there is a decrease in the entrainment of nucleocytoplasmic NAD^+^ levels and SIRT1 activity with the CLOCK-BMAL1 (ARNTL) heterodimer that controls circadian changes in gene expression. This causes decreased expression of the NAMPT gene and decreased NAD^+^ levels. With less nucleocytoplasmic NAD^+^ there is a decreased glycolytic rate, decreased NADK activity, decreased NADP(H) levels, increased oxidative damage, and continued progression of PD.

## 4. A Five-Pronged Strategy to Improve MQC for the Treatment of PD

Based on the experiences of the primary author of this article, who has lived with PD for the last 22 years and has been able to reverse his PD symptoms and maintain them at a low level for the past 7 years, a strategy has been devised to delay or even reverse PD progression, which is also hypothesized to improve neural MQC and increase neuronal NADPH levels. This strategy involves (1) avoiding carbohydrates and acute protein overfeeding both of which result in spikes in circulating insulin, and (2) short-term fasting (time-restricted feeding) from dinner until breakfast, which induces ketosis but does not employ a strict ketogenic diet. This may allow one to obtain mild ketosis of roughly 1 mM blood levels of BHB in the morning. The dietary therapy includes (3) consuming sugar-free coffee in the morning that includes coffee bean polyphenols, caffeine, and added fat in the form of grass-fed butter, grass-fed cream, and coconut oil. Caffeine has been shown to have benefits in PD models and its levels appear to decrease in humans as PD symptoms progress [291]. The next portion of the therapeutic intervention is (4) consumption of ketone ester in the morning, followed lastly by (5) fasted exercise directly following ketone ester consumption, which increases ADP levels and the NAD^+^/NADH and AMP/ATP ratios, which activate AMPK and autophagy. Increased ADP levels stimulate mitochondrial ETC activity including complex I, which oxidizes NADH to NAD^+^, which in turn leads to sirtuin activation increasing mitochondrial function and biogenesis. 

As mentioned earlier, exercise has also been shown to stimulate mitochondrial biogenesis in the brain [292,293]. The exercise session typically consists of 25 to 30 min of cycling, alternating high-intensity activity with recovery by adjusting the resistance. A period of ten minutes of stationary cycling on two to three days per week is followed by resistance training and stretching. The duration and intensity of the exercise should be modified according to what is appropriate for the age and the degree of symptoms of the PD subject, and could consist, for example, of walking, dancing, boxing, or physical therapy. Step 4 above, the ketone ester consumption, was added to a working protocol in the last couple of years to boost the BHB levels. This further decreased the PD symptoms to enable increased exercise intensity. However, some relief from symptoms was obtained in the absence of ketone ester consumption.

During an overnight fast, ketone bodies, glucagon, ghrelin [294], and orexin/hypocretin [295] increase, while insulin levels decline. These metabolic changes, together with morning fat consumption and exercise, stimulates the liver to produce BHB without requiring a ketogenic diet. Ketone bodies are known to possess many neuroprotective effects, such as increasing cytoplasmic NAD^+^ levels [296], activating the Nrf2 transcriptional regulator, and increasing antioxidant defense [297]. BHB also inhibits the NLRP3 inflammasome to decrease inflammation [298]. The combination of increased levels of cytoplasmic NAD^+^ and NADPH and increased cellular BHB (and possibly caffeine and coffee polyphenols) may allow for a beneficial “perfect storm” effect to stimulate mitochondrial biogenesis and turnover in both neurons and glia. The experience of the primary author suggests that the intervention requires all the individual parts of the therapy to be applied in synchrony to alleviate the severe PD symptoms. This intervention was designed to supplement and not to replace dopamine-targeted medications.

## 5. Conclusions

MQC becomes dysfunctional in PD and is a primary driver of the onset of disease and disease progression. When performed individually, fasting and exercise have been shown to have only minimal effects in the treatment of PD. However, combining an overnight and morning fast with morning exercise (and coffee consumption) may synergize with circadian oscillations in NAD^+^, NADPH, and mitophagy rates and be a promising therapy for PD. It is hypothesized that the increased levels of BHB, glucagon, ghrelin, and FGF21, and the decreased levels of insulin play major roles in providing the neuroprotection in this therapy. In addition to activating Nrf2 and possibly ATF4 to induce the expression of NADPH-synthesizing enzymes, BHB also activates neuroprotective pathways, inhibits proinflammatory mediators such as the NLRP3 inflammasome, and induces epigenetic effects that likely contribute to its neuroprotective effects. Clinical trials will aid in the determination of how efficacious this therapy is across the wide spectrum of sporadic and familial PD cases.

## References

[1] Panicker N., Ge P., Dawson V.L., Dawson T.M. (2021). The Cell Biology of Parkinson’s Disease. J. Cell Biol..

[2] Singh F., Ganley I.G. (2021). Parkinson’s Disease and Mitophagy: An Emerging Role for LRRK2. Biochem. Soc. Trans..

[3] Georgakopoulos N.D., Wells G., Campanella M. (2017). The Pharmacological Regulation of Cellular Mitophagy. Nat. Chem. Biol..

[4] Braak H., Del Tredici K., Rüb U., de Vos R.A.I., Jansen Steur E.N.H., Braak E. (2003). Staging of Brain Pathology Related to Sporadic Parkinson’s Disease. Neurobiol. Aging.

[5] Butkovich L.M., Houser M.C., Chalermpalanupap T., Porter-Stransky K.A., Iannitelli A.F., Boles J.S., Lloyd G.M., Coomes A.S., Eidson L.N., De Sousa Rodrigues M.E. (2020). Transgenic Mice Expressing Human α-Synuclein in Noradrenergic Neurons Develop Locus Ceruleus Pathology and Nonmotor Features of Parkinson’s Disease. J. Neurosci..

[6] Eldeeb M.A., Thomas R.A., Ragheb M.A., Fallahi A., Fon E.A. (2022). Mitochondrial Quality Control in Health and in Parkinson’s Disease. Physiol. Rev..

[7] Rafaeloff-Phail R., Ding L., Conner L., Yeh W.-K., McClure D., Guo H., Emerson K., Brooks H. (2004). Biochemical Regulation of Mammalian AMP-Activated Protein Kinase Activity by NAD and NADH. J. Biol. Chem..

[8] Egan D.F., Shackelford D.B., Mihaylova M.M., Gelino S., Kohnz R.A., Mair W., Vasquez D.S., Joshi A., Gwinn D.M., Taylor R. (2011). Phosphorylation of ULK1 (HATG1) by AMP-Activated Protein Kinase Connects Energy Sensing to Mitophagy. Science.

[9] Li W., Kou J., Qin J., Li L., Zhang Z., Pan Y., Xue Y., Du W. (2021). NADPH Levels Affect Cellular Epigenetic State by Inhibiting HDAC3–Ncor Complex. Nat. Metab..

[10] Sun X.-Y., Qu Y., Ni A.-R., Wang G.-X., Huang W.-B., Chen Z.-P., Lv Z.-F., Zhang S., Lindsay H., Zhao S. (2017). Novel Histone Deacetylase Inhibitor N25 Exerts Anti-Tumor Effects and Induces Autophagy in Human Glioma Cells by Inhibiting HDAC3. Oncotarget.

[11] Huang S., Chen G., Sun J., Chen Y., Wang N., Dong Y., Shen E., Hu Z., Gong W., Jin L. (2021). Histone Deacetylase 3 Inhibition Alleviates Type 2 Diabetes Mellitus-Induced Endothelial Dysfunction via Nrf2. Cell Commun. Signal..

[12] Vogelauer M., Krall A.S., McBrian M.A., Li J.-Y., Kurdistani S.K. (2012). Stimulation of Histone Deacetylase Activity by Metabolites of Intermediary Metabolism. J. Biol. Chem..

[13] Murari A., Goparaju N.S.V., Rhooms S.-K., Hossain K.F.B., Liang F.G., Garcia C.J., Osei C., Liu T., Li H., Kitsis R.N. (2022). IDH2-Mediated Regulation of the Biogenesis of the Oxidative Phosphorylation System. Sci. Adv..

[14] Abdrakhmanova A., Zwicker K., Kerscher S., Zickermann V., Brandt U. (2006). Tight Binding of NADPH to the 39-KDa Subunit of Complex I Is Not Required for Catalytic Activity but Stabilizes the Multiprotein Complex. Biochim. Biophys. Acta Bioenerg..

[15] McCarty M.F., Lerner A. (2020). Nutraceuticals Targeting Generation and Oxidant Activity of Peroxynitrite May Aid Prevention and Control of Parkinson’s Disease. Int. J. Mol. Sci..

[16] Rodriguez-Enriquez S., He L., Lemasters J.J. (2004). Role of Mitochondrial Permeability Transition Pores in Mitochondrial Autophagy. Int. J. Biochem. Cell Biol..

[17] Lemasters J.J., Nieminen A.-L., Qian T., Trost L.C., Elmore S.P., Nishimura Y., Crowe R.A., Cascio W.E., Bradham C.A., Brenner D.A. (1998). The Mitochondrial Permeability Transition in Cell Death: A Common Mechanism in Necrosis, Apoptosis and Autophagy. Biochim. Biophys. Acta Bioenerg..

[18] Salvi M., Battaglia V., Brunati A.M., Rocca N.L., Tibaldi E., Pietrangeli P., Marcocci L., Mondovi B., Rossi C.A., Toninello A. (2007). Catalase Takes Part in Rat Liver Mitochondria Oxidative Stress Defense. J. Biol. Chem..

[19] Radi R., Turrens J.F., Chang L.Y., Bush K.M., Crapo J.D., Freeman B.A. (1991). Detection of Catalase in Rat Heart Mitochondria. J. Biol. Chem..

[20] Menzies R.A., Gold P.H. (1971). The Turnover of Mitochondria in a Variety of Tissues of Young Adult and Aged Rats. J. Biol. Chem..

[21] Menzies R.A., Gold P.H. (1972). The Apparent Turnover of Mitochondria, Ribosomes and SRNA of the Brain in Young Adult and Aged Rats. J. Neurochem..

[22] Beattie D.S., Basford R.E., Koritz S.B. (1967). The Turnover of the Protein Components of Mitochondria from Rat Liver, Kidney, and Brain. J. Biol. Chem..

[23] Stotland A., Gottlieb R.A. (2015). Mitochondrial Quality Control: Easy Come, Easy Go. Biochim. Biophys. Acta Mol. Cell Res..

[24] Bomba-Warczak E., Edassery S.L., Hark T.J., Savas J.N. (2021). Long-Lived Mitochondrial Cristae Proteins in Mouse Heart and Brain. J. Cell Biol..

[25] Krishna S., Arrojo E Drigo R., Capitanio J.S., Ramachandra R., Ellisman M., Hetzer M.W. (2021). Identification of Long-Lived Proteins in the Mitochondria Reveals Increased Stability of the Electron Transport Chain. Dev. Cell.

[26] Twig G., Elorza A., Molina A.J.A., Mohamed H., Wikstrom J.D., Walzer G., Stiles L., Haigh S.E., Katz S., Las G. (2008). Fission and Selective Fusion Govern Mitochondrial Segregation and Elimination by Autophagy. EMBO J..

[27] Kluever V., Russo B., Mandad S., Kumar N.H., Alevra M., Ori A., Rizzoli S.O., Urlaub H., Schneider A., Fornasiero E.F. (2022). Protein Lifetimes in Aged Brains Reveal a Proteostatic Adaptation Linking Physiological Aging to Neurodegeneration. Sci. Adv..

[28] Misgeld T., Schwarz T.L. (2017). Mitostasis in Neurons: Maintaining Mitochondria in an Extended Cellular Architecture. Neuron.

[29] Lamberts J.T., Hildebrandt E.N., Brundin P. (2015). Spreading of α-Synuclein in the Face of Axonal Transport Deficits in Parkinson’s Disease: A Speculative Synthesis. Neurobiol. Dis..

[30] Gomez-Fabra Gala M., Vögtle F. (2021). Mitochondrial Proteases in Human Diseases. FEBS Lett..

[31] Chen Y., Azad M.B., Gibson S.B. (2009). Superoxide Is the Major Reactive Oxygen Species Regulating Autophagy. Cell Death Differ..

[32] Bandopadhyay R., Kingsbury A.E., Cookson M.R., Reid A.R., Evans I.M., Hope A.D., Pittman A.M., Lashley T., Canet-Aviles R., Miller D.W. (2004). The Expression of DJ-1 (PARK7) in Normal Human CNS and Idiopathic Parkinson’s Disease. Brain.

[33] Blackinton J., Lakshminarasimhan M., Thomas K.J., Ahmad R., Greggio E., Raza A.S., Cookson M.R., Wilson M.A. (2009). Formation of a Stabilized Cysteine Sulfinic Acid Is Critical for the Mitochondrial Function of the Parkinsonism Protein DJ-1. J. Biol. Chem..

[34] Holmström K.M., Kostov R.V., Dinkova-Kostova A.T. (2016). The Multifaceted Role of Nrf2 in Mitochondrial Function. Curr. Opin. Toxicol..

[35] Yao W., Lin S., Su J., Cao Q., Chen Y., Chen J., Zhang Z., Hashimoto K., Qi Q., Zhang J. (2021). Activation of BDNF by Transcription Factor Nrf2 Contributes to Antidepressant-like Actions in Rodents. Transl. Psychiatry.

[36] Dolgacheva L.P., Berezhnov A.V., Fedotova E.I., Zinchenko V.P., Abramov A.Y. (2019). Role of DJ-1 in the Mechanism of Pathogenesis of Parkinson’s Disease. J. Bioenerg. Biomembr..

[37] Guzman J.N., Sanchez-Padilla J., Wokosin D., Kondapalli J., Ilijic E., Schumacker P.T., Surmeier D.J. (2010). Oxidant Stress Evoked by Pacemaking in Dopaminergic Neurons Is Attenuated by DJ-1. Nature.

[38] Xu S., Yang X., Qian Y., Xiao Q. (2018). Parkinson’s Disease-Related DJ-1 Modulates the Expression of Uncoupling Protein 4 against Oxidative Stress. J. Neurochem..

[39] He C.H., Gong P., Hu B., Stewart D., Choi M.E., Choi A.M.K., Alam J. (2001). Identification of Activating Transcription Factor 4 (ATF4) as an Nrf2-Interacting Protein: Implication for Heme Oxygenase-1 Gene Regulation. J. Biol. Chem..

[40] Zong Z.-H., Du Z.-X., Li N., Li C., Zhang Q., Liu B.-Q., Guan Y., Wang H.-Q. (2012). Implication of Nrf2 and ATF4 in Differential Induction of CHOP by Proteasome Inhibition in Thyroid Cancer Cells. Biochim. Biophys. Acta Mol. Cell Res..

[41] Almeida L.M., Pinho B.R., Duchen M.R., Oliveira J.M.A. (2022). The PERKs of Mitochondria Protection during Stress: Insights for PERK Modulation in Neurodegenerative and Metabolic Diseases. Biol. Rev. Camb. Philos. Soc..

[42] Lange P.S., Chavez J.C., Pinto J.T., Coppola G., Sun C.-W., Townes T.M., Geschwind D.H., Ratan R.R. (2008). ATF4 Is an Oxidative Stress–Inducible, Prodeath Transcription Factor in Neurons in Vitro and in Vivo. J. Exp. Med..

[43] Quirós P.M., Prado M.A., Zamboni N., D’Amico D., Williams R.W., Finley D., Gygi S.P., Auwerx J. (2017). Multi-Omics Analysis Identifies ATF4 as a Key Regulator of the Mitochondrial Stress Response in Mammals. J. Cell Biol..

[44] Kasai S., Yamazaki H., Tanji K., Engler M.J., Matsumiya T., Itoh K. (2019). Role of the ISR-ATF4 Pathway and Its Cross Talk with Nrf2 in Mitochondrial Quality Control. J. Clin. Biochem. Nutr..

[45] Torrence M.E., MacArthur M.R., Hosios A.M., Valvezan A.J., Asara J.M., Mitchell J.R., Manning B.D. (2021). The MTORC1-Mediated Activation of ATF4 Promotes Protein and Glutathione Synthesis Downstream of Growth Signals. eLife.

[46] Renz P.F., Valdivia-Francia F., Sendoel A. (2020). Some like It Translated: Small ORFs in the 5′UTR. Exp. Cell Res..

[47] Endo J., Sano M., Katayama T., Hishiki T., Shinmura K., Morizane S., Matsuhashi T., Katsumata Y., Zhang Y., Ito H. (2009). Metabolic Remodeling Induced by Mitochondrial Aldehyde Stress Stimulates Tolerance to Oxidative Stress in the Heart. Circ. Res..

[48] Wang X., Zhang G., Dasgupta S., Niewold E.L., Li C., Li Q., Luo X., Tan L., Ferdous A., Lorenzi P.L. (2022). ATF4 Protects the Heart From Failure by Antagonizing Oxidative Stress. Circ. Res..

[49] Ben-Sahra I., Hoxhaj G., Ricoult S.J.H., Asara J.M., Manning B.D. (2016). MTORC1 Induces Purine Synthesis Through Control of the Mitochondrial Tetrahydrofolate Cycle. Science.

[50] Hill C.M., Albarado D.C., Coco L.G., Spann R.A., Khan M.S., Qualls-Creekmore E., Burk D.H., Burke S.J., Collier J.J., Yu S. (2022). FGF21 Is Required for Protein Restriction to Extend Lifespan and Improve Metabolic Health in Male Mice. Nat. Commun..

[51] Zhang Y., Xie Y., Berglund E.D., Coate K.C., He T.T., Katafuchi T., Xiao G., Potthoff M.J., Wei W., Wan Y. (2012). The Starvation Hormone, Fibroblast Growth Factor-21, Extends Lifespan in Mice. eLife.

[52] Klaus S., Igual Gil C., Ost M. (2021). Regulation of Diurnal Energy Balance by Mitokines. Cell Mol. Life Sci..

[53] Kim K.H., Kim S.H., Min Y.-K., Yang H.-M., Lee J.-B., Lee M.-S. (2013). Acute Exercise Induces FGF21 Expression in Mice and in Healthy Humans. PLoS ONE.

[54] Wan X., Lu X., Xiao Y., Lin Y., Zhu H., Ding T., Yang Y., Huang Y., Zhang Y., Liu Y.-L. (2014). ATF4- and CHOP-Dependent Induction of FGF21 through Endoplasmic Reticulum Stress. BioMed Res. Int..

[55] Bouman L., Schlierf A., Lutz A.K., Shan J., Deinlein A., Kast J., Galehdar Z., Palmisano V., Patenge N., Berg D. (2011). Parkin Is Transcriptionally Regulated by ATF4: Evidence for an Interconnection between Mitochondrial Stress and ER Stress. Cell Death. Differ..

[56] Li W., Li X., Miller R.A. (2014). ATF4 Activity: A Common Feature Shared by Many Kinds of Slow-Aging Mice. Aging Cell.

[57] Koyanagi S., Hamdan A.M., Horiguchi M., Kusunose N., Okamoto A., Matsunaga N., Ohdo S. (2011). CAMP-Response Element (CRE)-Mediated Transcription by Activating Transcription Factor-4 (ATF4) Is Essential for Circadian Expression of the Period2 Gene. J. Biol. Chem..

[58] Pathak S.S., Liu D., Li T., de Zavalia N., Zhu L., Li J., Karthikeyan R., Alain T., Liu A.C., Storch K.-F. (2019). The EIF2α Kinase GCN2 Modulates Period and Rhythmicity of the Circadian Clock by Translational Control of Atf4. Neuron.

[59] Haigis M.C., Mostoslavsky R., Haigis K.M., Fahie K., Christodoulou D.C., Murphy A.J., Valenzuela D.M., Yancopoulos G.D., Karow M., Blander G. (2006). SIRT4 Inhibits Glutamate Dehydrogenase and Opposes the Effects of Calorie Restriction in Pancreatic β Cells. Cell.

[60] Shaw E., Talwadekar M., Rashida Z., Mohan N., Acharya A., Khatri S., Laxman S., Kolthur-Seetharam U. (2020). Anabolic SIRT4 Exerts Retrograde Control over TORC1 Signaling by Glutamine Sparing in the Mitochondria. Mol. Cell Biol..

[61] de Goede P., Wüst R.C.I., Schomakers B.V., Denis S., Vaz F.M., Pras-Raves M.L., van Weeghel M., Yi C.-X., Kalsbeek A., Houtkooper R.H. (2022). Time-Restricted Feeding during the Inactive Phase Abolishes the Daily Rhythm in Mitochondrial Respiration in Rat Skeletal Muscle. FASEB J..

[62] Chin R.M., Fu X., Pai M.Y., Vergnes L., Hwang H., Deng G., Diep S., Lomenick B., Meli V.S., Monsalve G.C. (2014). The Metabolite Alpha-Ketoglutarate Extends Lifespan by Inhibiting the ATP Synthase and TOR. Nature.

[63] Su Y., Wang T., Wu N., Li D., Fan X., Xu Z., Mishra S.K., Yang M. (2019). Alpha-Ketoglutarate Extends Drosophila Lifespan by Inhibiting MTOR and Activating AMPK. Aging.

[64] Shahmirzadi A.A., Edgar D., Liao C.-Y., Hsu Y.-M., Lucanic M., Shahmirzadi A.A., Wiley C.D., Gan G., Kim D.E., Kasler H.G. (2020). Alpha-Ketoglutarate, an Endogenous Metabolite, Extends Lifespan and Compresses Morbidity in Aging Mice. Cell Metab..

[65] Harrison A.P., Pierzynowski S.G. (2008). Biological Effects of 2-Oxoglutarate with Particular Emphasis on the Regulation of Protein, Mineral and Lipid Absorption/Metabolism, Muscle Performance, Kidney Function, Bone Formation and Cancerogenesis, All Viewed from a Healthy Ageing Perspective State of the Art—Review Article. J. Physiol. Pharmacol..

[66] Lin A.-L., Zhang W., Gao X., Watts L. (2015). Caloric Restriction Increases Ketone Bodies Metabolism and Preserves Blood Flow in Aging Brain. Neurobiol. Aging.

[67] Shrimali N.M., Agarwal S., Kaur S., Bhattacharya S., Bhattacharyya S., Prchal J.T., Guchhait P. (2021). α-Ketoglutarate Inhibits Thrombosis and Inflammation by Prolyl Hydroxylase-2 Mediated Inactivation of Phospho-Akt. eBioMedicine.

[68] Wang Y., Deng P., Liu Y., Wu Y., Chen Y., Guo Y., Zhang S., Zheng X., Zhou L., Liu W. (2020). Alpha-Ketoglutarate Ameliorates Age-Related Osteoporosis via Regulating Histone Methylations. Nat. Commun..

[69] Satpute R., Lomash V., Kaushal M., Bhattacharya R. (2013). Neuroprotective Effects of Alpha-Ketoglutarate and Ethyl Pyruvate against Motor Dysfunction and Oxidative Changes Caused by Repeated 1-Methyl-4-Phenyl-1,2,3,6 Tetrahydropyridine Exposure in Mice. Hum. Exp. Toxicol..

[70] Yang Y.R., Kwon K.-S. (2020). Potential Roles of Exercise-Induced Plasma Metabolites Linking Exercise to Health Benefits. Front. Physiol..

[71] Daneshmandi S., Cassel T., Higashi R.M., Fan T.W.-M., Seth P. (2021). 6-Phosphogluconate Dehydrogenase (6PGD), a Key Checkpoint in Reprogramming of Regulatory T Cells Metabolism and Function. eLife.

[72] Liu Q., Zhu F., Liu X., Lu Y., Yao K., Tian N., Tong L., Figge D.A., Wang X., Han Y. (2022). Non-Oxidative Pentose Phosphate Pathway Controls Regulatory T Cell Function by Integrating Metabolism and Epigenetics. Nat. Metab..

[73] Moebus S., Göres L., Lösch C., Jöckel K.-H. (2011). Impact of Time since Last Caloric Intake on Blood Glucose Levels. Eur. J. Epidemiol..

[74] Gannon M.C., Nuttall F.Q., Lane J.T., Fang S., Gupta V., Sandhofer C.R. (1996). Effect of 24 Hours of Starvation on Plasma Glucose and Insulin Concentrations in Subjects with Untreated Non—Insulin-Dependent Diabetes Mellitus. Metabolism.

[75] Owen O.E., Felig P., Morgan A.P., Wahren J., Cahill G.F. (1969). Liver and Kidney Metabolism during Prolonged Starvation. J. Clin. Investig..

[76] Cahill G.F. (2006). Fuel Metabolism in Starvation. Annu. Rev. Nutr..

[77] Minich T., Yokota S., Dringen R. (2003). Cytosolic and Mitochondrial Isoforms of NADP^+^-Dependent Isocitrate Dehydrogenases Are Expressed in Cultured Rat Neurons, Astrocytes, Oligodendrocytes and Microglial Cells. J. Neurochem..

[78] Kurz G.M., Wiesinger H., Hamprecht B. (1993). Purification of Cytosolic Malic Enzyme from Bovine Brain, Generation of Monoclonal Antibodies, and Immunocytochemical Localization of the Enzyme in Glial Cells of Neural Primary Cultures. J. Neurochem..

[79] Jiménez-Jiménez F.J., Alonso-Navarro H., García-Martín E., Agúndez J.A.G. (2020). Cerebrospinal and Blood Levels of Amino Acids as Potential Biomarkers for Parkinson’s Disease: Review and Meta-analysis. Eur. J. Neurol..

[80] Stanton R.C. (2012). Glucose-6-Phosphate Dehydrogenase, NADPH, and Cell Survival. IUBMB Life.

[81] Zhang Z., TeSlaa T., Xu X., Zeng X., Yang L., Xing G., Tesz G.J., Clasquin M.F., Rabinowitz J.D. (2021). Serine Catabolism Generates Liver NADPH and Supports Hepatic Lipogenesis. Nat. Metab..

[82] Veech R.L., Eggleston L.V., Krebs H.A. (1969). The Redox State of Free Nicotinamide–Adenine Dinucleotide Phosphate in the Cytoplasm of Rat Liver. Biochem. J..

[83] Wasselin T., Zahn S., Maho Y.L., Dorsselaer A.V., Raclot T., Bertile F. (2014). Exacerbated Oxidative Stress in the Fasting Liver According to Fuel Partitioning. Proteomics.

[84] Pawlosky R.J., Kemper M.F., Kashiwaya Y., King M.T., Mattson M.P., Veech R.L. (2017). Effects of a Dietary Ketone Ester on Hippocampal Glycolytic and TCA Cycle Intermediates and Amino Acids in a 3xTgAD Mouse Model of Alzheimer’s Disease. J. Neurochem..

[85] Someya S., Yu W., Hallows W.C., Xu J., Vann J.M., Leeuwenburgh C., Tanokura M., Denu J.M., Prolla T.A. (2010). Sirt3 Mediates Reduction of Oxidative Damage and Prevention of Age-Related Hearing Loss under Caloric Restriction. Cell.

[86] Mary C., Soflaee M.H., Kesavan R., Gelin M., Brown H., Zacharias G., Mathews T.P., Lemoff A., Lionne C., Labesse G. Crystal Structure of Human NADK2 Reveals a Dimeric Organization and Active Site Occlusion by Lysine Acetylation. Mol. Cell.

[87] Verma D.K., Seo B.A., Ghosh A., Ma S.-X., Hernandez-Quijada K., Andersen J.K., Ko H.S., Kim Y.-H. (2021). Alpha-Synuclein Preformed Fibrils Induce Cellular Senescence in Parkinson’s Disease Models. Cells.

[88] Chinta S.J., Woods G., Demaria M., Rane A., Zou Y., McQuade A., Rajagopalan S., Limbad C., Madden D.T., Campisi J. (2018). Cellular Senescence Is Induced by the Environmental Neurotoxin Paraquat and Contributes to Neuropathology Linked to Parkinson’s Disease. Cell Rep..

[89] Martini H., Passos J.F. (2022). Cellular Senescence: All Roads Lead to Mitochondria. FEBS J..

[90] Zhao Y., Liu B., Xu L., Yu S., Fu J., Wang J., Yan X., Su J. (2021). ROS-Induced MtDNA Release: The Emerging Messenger for Communication between Neurons and Innate Immune Cells during Neurodegenerative Disorder Progression. Antioxidants.

[91] Xian H., Watari K., Sanchez-Lopez E., Offenberger J., Onyuru J., Sampath H., Ying W., Hoffman H.M., Shadel G.S., Karin M. Oxidized DNA Fragments Exit Mitochondria via MPTP- and VDAC-Dependent Channels to Activate NLRP3 Inflammasome and Interferon Signaling. Immunity.

[92] Salnikov V., Lukyánenko Y.O., Frederick C.A., Lederer W.J., Lukyánenko V. (2007). Probing the Outer Mitochondrial Membrane in Cardiac Mitochondria with Nanoparticles. Biophys. J..

[93] Rauckhorst A.J., Pfeiffer D.R., Broekemeier K.M. (2015). The IPLA2γ Is Identified as the Membrane Potential Sensitive Phospholipase in Liver Mitochondria. FEBS Lett..

[94] Patrushev M., Kasymov V., Patrusheva V., Ushakova T., Gogvadze V., Gaziev A. (2004). Mitochondrial Permeability Transition Triggers the Release of MtDNA Fragments. CMLS Cell. Mol. Life Sci..

[95] García N., García J.J., Correa F., Chávez E. (2005). The Permeability Transition Pore as a Pathway for the Release of Mitochondrial DNA. Life Sci..

[96] Hou Y., Wei Y., Lautrup S., Yang B., Wang Y., Cordonnier S., Mattson M.P., Croteau D.L., Bohr V.A. (2021). NAD+ Supplementation Reduces Neuroinflammation and Cell Senescence in a Transgenic Mouse Model of Alzheimer’s Disease via CGAS–STING. Proc. Natl. Acad. Sci. USA.

[97] Bender A., Krishnan K.J., Morris C.M., Taylor G.A., Reeve A.K., Perry R.H., Jaros E., Hersheson J.S., Betts J., Klopstock T. (2006). High Levels of Mitochondrial DNA Deletions in Substantia Nigra Neurons in Aging and Parkinson Disease. Nat. Genet..

[98] Kraytsberg Y., Kudryavtseva E., McKee A.C., Geula C., Kowall N.W., Khrapko K. (2006). Mitochondrial DNA Deletions Are Abundant and Cause Functional Impairment in Aged Human Substantia Nigra Neurons. Nat. Genet..

[99] Dölle C., Flønes I., Nido G.S., Miletic H., Osuagwu N., Kristoffersen S., Lilleng P.K., Larsen J.P., Tysnes O.-B., Haugarvoll K. (2016). Defective Mitochondrial DNA Homeostasis in the Substantia Nigra in Parkinson Disease. Nat. Commun..

[100] Manini A., Abati E., Comi G.P., Corti S., Ronchi D. (2022). Mitochondrial DNA Homeostasis Impairment and Dopaminergic Dysfunction: A Trembling Balance. Ageing Res. Rev..

[101] Wiley C.D., Velarde M.C., Lecot P., Liu S., Sarnoski E.A., Freund A., Shirakawa K., Lim H.W., Davis S.S., Ramanathan A. (2016). Mitochondrial Dysfunction Induces Senescence with a Distinct Secretory Phenotype. Cell Metab..

[102] Kujoth G.C., Hiona A., Pugh T.D., Someya S., Panzer K., Wohlgemuth S.E., Hofer T., Seo A.Y., Sullivan R., Jobling W.A. (2005). Mitochondrial DNA Mutations, Oxidative Stress, and Apoptosis in Mammalian Aging. Science.

[103] Tyynismaa H., Mjosund K.P., Wanrooij S., Lappalainen I., Ylikallio E., Jalanko A., Spelbrink J.N., Paetau A., Suomalainen A. (2005). Mutant Mitochondrial Helicase Twinkle Causes Multiple MtDNA Deletions and a Late-Onset Mitochondrial Disease in Mice. Proc. Natl. Acad. Sci. USA.

[104] Vermulst M., Wanagat J., Kujoth G.C., Bielas J.H., Rabinovitch P.S., Prolla T.A., Loeb L.A. (2008). DNA Deletions and Clonal Mutations Drive Premature Aging in Mitochondrial Mutator Mice. Nat. Genet..

[105] Someya S., Kujoth G.C., Kim M.-J., Hacker T.A., Vermulst M., Weindruch R., Prolla T.A. (2017). Effects of Calorie Restriction on the Lifespan and Healthspan of POLG Mitochondrial Mutator Mice. PLoS ONE.

[106] Colman R.J., Beasley T.M., Kemnitz J.W., Johnson S.C., Weindruch R., Anderson R.M. (2014). Caloric Restriction Reduces Age-Related and All-Cause Mortality in Rhesus Monkeys. Nat. Commun..

[107] McKiernan S.H., Colman R.J., Aiken E., Evans T.D., Beasley T.M., Aiken J.M., Weindruch R., Anderson R.M. (2012). Cellular Adaptation Contributes to Calorie Restriction-Induced Preservation of Skeletal Muscle in Aged Rhesus Monkeys. Exp. Gerontol..

[108] Finley L.W.S., Lee J., Souza A., Desquiret-Dumas V., Bullock K., Rowe G.C., Procaccio V., Clish C.B., Arany Z., Haigis M.C. (2012). Skeletal Muscle Transcriptional Coactivator PGC-1α Mediates Mitochondrial, but Not Metabolic, Changes during Calorie Restriction. Proc. Natl. Acad. Sci. USA.

[109] Guo X., Kudryavtseva E., Bodyak N., Nicholas A., Dombrovsky I., Yang D., Kraytsberg Y., Simon D.K., Khrapko K. (2010). Mitochondrial DNA Deletions in Mice in Men: Substantia Nigra Is Much Less Affected in the Mouse. Biochim. Biophys. Acta.

[110] Dai Y., Kiselak T., Clark J., Clore E., Zheng K., Cheng A., Kujoth G.C., Prolla T.A., Maratos-Flier E., Simon D.K. (2013). Behavioral and Metabolic Characterization of Heterozygous and Homozygous POLG Mutator Mice. Mitochondrion.

[111] Green C.L., Lamming D.W., Fontana L. (2022). Molecular Mechanisms of Dietary Restriction Promoting Health and Longevity. Nat. Rev. Mol. Cell Biol..

[112] Mitchell S.J., Bernier M., Mattison J.A., Aon M.A., Kaiser T.A., Anson R.M., Ikeno Y., Anderson R.M., Ingram D.K., de Cabo R. (2019). Daily Fasting Improves Health and Survival in Male Mice Independent of Diet Composition and Calories. Cell Metab..

[113] Shimokawa I., Komatsu T., Hayashi N., Kim S.-E., Kawata T., Park S., Hayashi H., Yamaza H., Chiba T., Mori R. (2015). The Life-Extending Effect of Dietary Restriction Requires Foxo3 in Mice. Aging Cell.

[114] Edwards C., Canfield J., Copes N., Rehan M., Lipps D., Bradshaw P.C. (2014). D-Beta-Hydroxybutyrate Extends Lifespan in *C. elegans*. Aging.

[115] Mattson M.P., Moehl K., Ghena N., Schmaedick M., Cheng A. (2018). Intermittent Metabolic Switching, Neuroplasticity and Brain Health. Nat. Rev. Neurosci..

[116] Hvingelby V.S., Glud A.N., Sørensen J.C.H., Tai Y., Andersen A.S.M., Johnsen E., Moro E., Pavese N. (2022). Interventions to Improve Gait in Parkinson’s Disease: A Systematic Review of Randomized Controlled Trials and Network Meta-Analysis. J. Neurol..

[117] Uhrbrand A., Stenager E., Pedersen M.S., Dalgas U. (2015). Parkinson’s Disease and Intensive Exercise Therapy—A Systematic Review and Meta-Analysis of Randomized Controlled Trials. J. Neurol. Sci..

[118] Stillman C.M., Cohen J., Lehman M.E., Erickson K.I. (2016). Mediators of Physical Activity on Neurocognitive Function: A Review at Multiple Levels of Analysis. Front. Hum. Neurosci..

[119] Petzinger G.M., Walsh J.P., Akopian G., Hogg E., Abernathy A., Arevalo P., Turnquist P., Vučković M., Fisher B.E., Togasaki D.M. (2007). Effects of Treadmill Exercise on Dopaminergic Transmission in the 1-Methyl-4-Phenyl-1,2,3,6-Tetrahydropyridine-Lesioned Mouse Model of Basal Ganglia Injury. J. Neurosci..

[120] Stranahan A.M., Lee K., Martin B., Maudsley S., Golden E., Cutler R.G., Mattson M.P. (2009). Voluntary Exercise and Caloric Restriction Enhance Hippocampal Dendritic Spine Density and BDNF Levels in Diabetic Mice. Hippocampus.

[121] Lee J., Duan W., Mattson M.P. (2002). Evidence That Brain-Derived Neurotrophic Factor Is Required for Basal Neurogenesis and Mediates, in Part, the Enhancement of Neurogenesis by Dietary Restriction in the Hippocampus of Adult Mice. J. Neurochem..

[122] Vivar C., Potter M.C., Choi J., Lee J., Stringer T.P., Callaway E.M., Gage F.H., Suh H., van Praag H. (2012). Monosynaptic Inputs to New Neurons in the Dentate Gyrus. Nat. Commun..

[123] Estrada N.M., Isokawa M. (2009). Metabolic Demand Stimulates CREB Signaling in the Limbic Cortex: Implication for the Induction of Hippocampal Synaptic Plasticity by Intrinsic Stimulus for Survival. Front. Syst. Neurosci..

[124] Yang J.-L., Lin Y.-T., Chuang P.-C., Bohr V.A., Mattson M.P. (2014). BDNF and Exercise Enhance Neuronal DNA Repair by Stimulating CREB-Mediated Production of Apurinic/Apyrimidinic Endonuclease 1. Neuromol. Med..

[125] Marosi K., Mattson M.P. (2014). BDNF Mediates Adaptive Brain and Body Responses to Energetic Challenges. Trends Endocrinol. Metab. TEM.

[126] Marosi K., Kim S.W., Moehl K., Scheibye-Knudsen M., Cheng A., Cutler R., Camandola S., Mattson M.P. (2016). 3-Hydroxybutyrate Regulates Energy Metabolism and Induces BDNF Expression in Cerebral Cortical Neurons. J. Neurochem..

[127] Sleiman S.F., Henry J., Al-Haddad R., El Hayek L., Abou Haidar E., Stringer T., Ulja D., Karuppagounder S.S., Holson E.B., Ratan R.R. (2016). Exercise Promotes the Expression of Brain Derived Neurotrophic Factor (BDNF) through the Action of the Ketone Body β-Hydroxybutyrate. eLife.

[128] Hood D.A., Tryon L.D., Carter H.N., Kim Y., Chen C.C.W. (2016). Unravelling the Mechanisms Regulating Muscle Mitochondrial Biogenesis. Biochem. J..

[129] Di Benedetto S., Müller L., Wenger E., Düzel S., Pawelec G. (2017). Contribution of Neuroinflammation and Immunity to Brain Aging and the Mitigating Effects of Physical and Cognitive Interventions. Neurosci. Biobehav. Rev..

[130] Simeone T.A., Simeone K.A., Rho J.M. (2017). Ketone Bodies as Anti-Seizure Agents. Neurochem. Res..

[131] Ngandu T., Lehtisalo J., Solomon A., Levälahti E., Ahtiluoto S., Antikainen R., Bäckman L., Hänninen T., Jula A., Laatikainen T. (2015). A 2 Year Multidomain Intervention of Diet, Exercise, Cognitive Training, and Vascular Risk Monitoring versus Control to Prevent Cognitive Decline in at-Risk Elderly People (FINGER): A Randomised Controlled Trial. Lancet.

[132] Grammatikopoulou M.G., Tousinas G., Balodimou C., Anastasilakis D.A., Gkiouras K., Dardiotis E., Evangeliou A.E., Bogdanos D.P., Goulis D.G. (2022). Ketogenic Therapy for Parkinson’s Disease: A Systematic Review and Synthesis without Meta-Analysis of Animal and Human Trials. Maturitas.

[133] Choi A., Hallett M., Ehrlich D. (2021). Nutritional Ketosis in Parkinson’s Disease—A Review of Remaining Questions and Insights. Neurotherapeutics.

[134] Kwon J.H., Moon K.M., Min K.-W. (2020). Exercise-Induced Myokines Can Explain the Importance of Physical Activity in the Elderly: An Overview. Healthcare.

[135] Hao Z., Zhang X., Chen P. (2022). Effects of Ten Different Exercise Interventions on Motor Function in Parkinson’s Disease Patients—A Network Meta-Analysis of Randomized Controlled Trials. Brain Sci..

[136] Bindoff L.A., Birch-Machin M.A., Cartlidge N.E.F., Parker W.D., Turnbull D.M. (1991). Respiratory Chain Abnormalities in Skeletal Muscle from Patients with Parkinson’s Disease. J. Neurol. Sci..

[137] Blin O., Desnuelle C., Rascol O., Borg M., Peyro Saint Paul H., Azulay J.P., Billé F., Figarella D., Coulom F., Pellissier J.F. (1994). Mitochondrial Respiratory Failure in Skeletal Muscle from Patients with Parkinson’s Disease and Multiple System Atrophy. J. Neurol. Sci..

[138] Cardellach F., Martí M.J., Fernández-Solá J., Marín C., Hoek J.B., Tolosa E., Urbano-Márquez A. (1993). Mitochondrial Respiratory Chain Activity in Skeletal Muscle from Patients with Parkinson’s Disease. Neurology.

[139] Mann V.M., Cooper J.M., Kridge D., Daniel S.E., Schapira A.H.V., Marsden C.D. (1992). Brain, Skeletal Muscle and Platelet Homogenate Mitochondrial Function in Parkinson’s Disease. Brain.

[140] Harper C., Gopalan V., Goh J. (2021). Exercise Rescues Mitochondrial Coupling in Aged Skeletal Muscle: A Comparison of Different Modalities in Preventing Sarcopenia. J. Transl. Med..

[141] Erlich A.T., Brownlee D.M., Beyfuss K., Hood D.A. (2018). Exercise Induces TFEB Expression and Activity in Skeletal Muscle in a PGC-1α-Dependent Manner. Am. J. Physiol. Cell Physiol..

[142] Gendi F., Pei F., Wang Y., Li H., Fu J., Chang C. (2022). Mitochondrial Proteins Unveil the Mechanism by Which Physical Exercise Ameliorates Memory, Learning and Motor Activity in Hypoxic Ischemic Encephalopathy Rat Model. Int. J. Mol. Sci..

[143] Mesquita P.H.C., Lamb D.A., Parry H.A., Moore J.H., Smith M.A., Vann C.G., Osburn S.C., Fox C.D., Ruple B.A., Huggins K.W. (2020). Acute and Chronic Effects of Resistance Training on Skeletal Muscle Markers of Mitochondrial Remodeling in Older Adults. Physiol. Rep..

[144] Lamb D.A., Moore J.H., Mesquita P.H.C., Smith M.A., Vann C.G., Osburn S.C., Fox C.D., Lopez H.L., Ziegenfuss T.N., Huggins K.W. (2020). Resistance Training Increases Muscle NAD+ and NADH Concentrations as Well as NAMPT Protein Levels and Global Sirtuin Activity in Middle-Aged, Overweight, Untrained Individuals. Aging.

[145] Wang L., Mascher H., Psilander N., Blomstrand E., Sahlin K. (2011). Resistance Exercise Enhances the Molecular Signaling of Mitochondrial Biogenesis Induced by Endurance Exercise in Human Skeletal Muscle. J. Appl. Physiol..

[146] Gibala M.J., Little J.P., MacDonald M.J., Hawley J.A. (2012). Physiological Adaptations to Low-Volume, High-Intensity Interval Training in Health and Disease. J. Physiol..

[147] Wahl P., Bloch W., Proschinger S. (2022). The Molecular Signature of High-Intensity Training in the Human Body. Int. J. Sports Med..

[148] Ma J.K., Scribbans T.D., Edgett B.A., Boyd J.C., Simpson C.A., Little J.P., Gurd B.J. (2013). Extremely Low-Volume, High-Intensity Interval Training Improves Exercise Capacity and Increases Mitochondrial Protein Content in Human Skeletal Muscle. Open J. Mol. Integr. Physiol..

[149] Santiago J.A., Quinn J.P., Potashkin J.A. (2022). Physical Activity Rewires the Human Brain against Neurodegeneration. Int. J. Mol. Sci..

[150] Palma J.-A., Kaufmann H. (2018). Treatment of Autonomic Dysfunction in Parkinson Disease and Other Synucleinopathies. Mov. Disord..

[151] Pfeiffer R.F. (2020). Autonomic Dysfunction in Parkinson’s Disease. Neurotherapeutics.

[152] Nalls M.A., McLean C.Y., Rick J., Eberly S., Hutten S.J., Gwinn K., Sutherland M., Martinez M., Heutink P., Williams N. (2015). Diagnosis of Parkinson’s Disease on the Basis of Clinical–Genetic Classification: A Population-Based Modelling Study. Lancet Neurol..

[153] De Abreu R.M., Rehder-Santos P., Simões R.P., Catai A.M. (2019). Can High-Intensity Interval Training Change Cardiac Autonomic Control? A Systematic Review. Braz. J. Phys. Ther..

[154] Harvey M., Weston K.L., Gray W.K., O’Callaghan A., Oates L.L., Davidson R., Walker R.W. (2019). High-Intensity Interval Training in People with Parkinson’s Disease: A Randomized, Controlled Feasibility Trial. Clin. Rehabil..

[155] Sato S., Dyar K.A., Treebak J.T., Jepsen S.L., Ehrlich A.M., Ashcroft S.P., Trost K., Kunzke T., Prade V.M., Small L. (2022). Atlas of Exercise Metabolism Reveals Time-Dependent Signatures of Metabolic Homeostasis. Cell Metab..

[156] Williamson D.H., Lund P., Krebs H.A. (1967). The Redox State of Free Nicotinamide-Adenine Dinucleotide in the Cytoplasm and Mitochondria of Rat Liver. Biochem. J..

[157] Goodman R.P., Markhard A.L., Shah H., Sharma R., Skinner O.S., Clish C.B., Deik A., Patgiri A., Hsu Y.-H., Masia R. (2020). Hepatic NADH Reductive Stress Underlies Common Variation in Metabolic Traits. Nature.

[158] Naghizadeh F. (1977). Oxidation of Alpha-Hydroxybutyrate by Human Serum. Clin. Chim. Acta.

[159] Lu S.C. (2013). Glutathione Synthesis. Biochim. Biophys. Acta Gen. Subj..

[160] Hine C., Harputlugil E., Zhang Y., Ruckenstuhl C., Lee B.C., Brace L., Longchamp A., Trevino-Villarreal J.H., Mejia P., Ozaki C.K. (2015). Endogenous Hydrogen Sulfide Production Is Essential for Dietary Restriction Benefits. Cell.

[161] Acosta-Rodríguez V., Rijo-Ferreira F., Izumo M., Xu P., Wight-Carter M., Green C.B., Takahashi J.S. (2022). Circadian Alignment of Early Onset Caloric Restriction Promotes Longevity in Male C57BL/6J Mice. Science.

[162] Peluso A., Damgaard M.V., Mori M.A.S., Treebak J.T. (2021). Age-Dependent Decline of NAD+—Universal Truth or Confounded Consensus?. Nutrients.

[163] Conlon N., Ford D. (2022). A Systems-Approach to NAD+ Restoration. Biochem. Pharmacol..

[164] Zeidler J.D., Hogan K.A., Agorrody G., Peclat T.R., Kashyap S., Kanamori K.S., Gomez L.S., Mazdeh D.Z., Warner G.M., Thompson K.L. (2022). The CD38 Glycohydrolase and the NAD Sink: Implications for Pathological Conditions. Am. J. Physiol. Cell Physiol..

[165] Strømland Ø., Diab J., Ferrario E., Sverkeli L.J., Ziegler M. (2021). The Balance between NAD+ Biosynthesis and Consumption in Ageing. Mech. Ageing Dev..

[166] Reiten O.K., Wilvang M.A., Mitchell S.J., Hu Z., Fang E.F. (2021). Preclinical and Clinical Evidence of NAD+ Precursors in Health, Disease, and Ageing. Mech. Ageing Dev..

[167] Magni G., Orsomando G., Raffaelli N. (2006). Structural and Functional Properties of NAD Kinase, a Key Enzyme in NADP Biosynthesis. MRMC.

[168] Du J., Estrella M., Solorio-Kirpichyan K., Jeffrey P.D., Korennykh A. (2022). Structure of Human NADK2 Reveals Atypical Assembly and Regulation of NAD Kinases from Animal Mitochondria. Proc. Natl. Acad. Sci. USA.

[169] Murata K. (2021). Polyphosphate-Dependent Nicotinamide Adenine Dinucleotide (NAD) Kinase: A Novel Missing Link in Human Mitochondria. Proc. Jpn Acad. Ser. B Phys. Biol. Sci..

[170] Veech R.L., Todd King M., Pawlosky R., Kashiwaya Y., Bradshaw P.C., Curtis W. (2019). The “Great” Controlling Nucleotide Coenzymes. IUBMB Life.

[171] Pollak N., Niere M., Ziegler M. (2007). NAD Kinase Levels Control the NADPH Concentration in Human Cells. J. Biol. Chem..

[172] Shats I., Williams J.G., Liu J., Makarov M.V., Wu X., Lih F.B., Deterding L.J., Lim C., Xu X., Randall T.A. (2020). Bacteria Boost Mammalian Host NAD Metabolism by Engaging the Deamidated Biosynthesis Pathway. Cell Metab..

[173] Covarrubias A.J., Perrone R., Grozio A., Verdin E. (2021). NAD+ Metabolism and Its Roles in Cellular Processes during Ageing. Nat. Rev. Mol. Cell. Biol..

[174] Liu L., Su X., Quinn W.J., Hui S., Krukenberg K., Frederick D.W., Redpath P., Zhan L., Chellappa K., White E. (2018). Quantitative Analysis of NAD Synthesis-Breakdown Fluxes. Cell Metab..

[175] Brakedal B., Dölle C., Riemer F., Ma Y., Nido G.S., Skeie G.O., Craven A.R., Schwarzlmüller T., Brekke N., Diab J. (2022). The NADPARK Study: A Randomized Phase I Trial of Nicotinamide Riboside Supplementation in Parkinson’s Disease. Cell Metab..

[176] Perrin L., Loizides-Mangold U., Chanon S., Gobet C., Hulo N., Isenegger L., Weger B.D., Migliavacca E., Charpagne A., Betts J.A. (2018). Transcriptomic Analyses Reveal Rhythmic and CLOCK-Driven Pathways in Human Skeletal Muscle. eLife.

[177] Schug T.T., Li X. (2011). Sirtuin 1 in Lipid Metabolism and Obesity. Ann. Med..

[178] Luna A., McFadden G.B., Aladjem M.I., Kohn K.W. (2015). Predicted Role of NAD Utilization in the Control of Circadian Rhythms during DNA Damage Response. PLoS Comput. Biol..

[179] Ramsey K.M., Yoshino J., Brace C.S., Abrassart D., Kobayashi Y., Marcheva B., Hong H.-K., Chong J.L., Buhr E.D., Lee C. (2009). Circadian Clock Feedback Cycle Through NAMPT-Mediated NAD+ Biosynthesis. Science.

[180] van Moorsel D., Hansen J., Havekes B., Scheer F.A.J.L., Jörgensen J.A., Hoeks J., Schrauwen-Hinderling V.B., Duez H., Lefebvre P., Schaper N.C. (2016). Demonstration of a Day-Night Rhythm in Human Skeletal Muscle Oxidative Capacity. Mol. Metab..

[181] Qiu Z., Ming H., Lei S., Zhou B., Zhao B., Yu Y., Xue R., Xia Z. (2021). Roles of HDAC3-Orchestrated Circadian Clock Gene Oscillations in Diabetic Rats Following Myocardial Ischaemia/Reperfusion Injury. Cell Death Dis..

[182] Goodman R.P., Calvo S.E., Mootha V.K. (2018). Spatiotemporal Compartmentalization of Hepatic NADH and NADPH Metabolism. J. Biol. Chem..

[183] Vallée A., Lecarpentier Y., Guillevin R., Vallée J.-N. (2020). Circadian Rhythms, Neuroinflammation and Oxidative Stress in the Story of Parkinson’s Disease. Cells.

[184] Hunt J., Coulson E.J., Rajnarayanan R., Oster H., Videnovic A., Rawashdeh O. (2022). Sleep and Circadian Rhythms in Parkinson’s Disease and Preclinical Models. Mol. Neurodegener..

[185] Prudon B., Duncan G.W., Khoo T.K., Yarnall A.J., Burn D.J., Anderson K.N. (2014). Primary Sleep Disorder Prevalence in Patients with Newly Diagnosed Parkinson’s Disease. Mov. Disord..

[186] Korshunov K.S., Blakemore L.J., Trombley P.Q. (2017). Dopamine: A Modulator of Circadian Rhythms in the Central Nervous System. Front. Cell. Neurosci..

[187] Mendoza J., Challet E. (2014). Circadian Insights into Dopamine Mechanisms. Neuroscience.

[188] Hood S., Cassidy P., Cossette M.-P., Weigl Y., Verwey M., Robinson B., Stewart J., Amir S. (2010). Endogenous Dopamine Regulates the Rhythm of Expression of the Clock Protein PER2 in the Rat Dorsal Striatum via Daily Activation of D2 Dopamine Receptors. J. Neurosci..

[189] Yujnovsky I., Hirayama J., Doi M., Borrelli E., Sassone-Corsi P. (2006). Signaling Mediated by the Dopamine D2 Receptor Potentiates Circadian Regulation by CLOCK:BMAL1. Proc. Natl. Acad. Sci. USA.

[190] Imbesi M., Yildiz S., Arslan A.D., Sharma R., Manev H., Uz T. (2009). Dopamine Receptor-Mediated Regulation of Neuronal “Clock” Gene Expression. Neuroscience.

[191] Parekh P.K., Ozburn A.R., McClung C.A. (2015). Circadian Clock Genes: Effects on Dopamine, Reward and Addiction. Alcohol.

[192] Breen D.P., Vuono R., Nawarathna U., Fisher K., Shneerson J.M., Reddy A.B., Barker R.A. (2014). Sleep and Circadian Rhythm Regulation in Early Parkinson Disease. JAMA Neurol..

[193] Cai Y., Liu S., Sothern R.B., Xu S., Chan P. (2010). Expression of Clock Genes *Per1* and *Bmal1* in Total Leukocytes in Health and Parkinson’s Disease: Clock Genes in PD. Eur. J. Neurol..

[194] Li S.-Y., Wang Y.-L., Liu W.-W., Lyu D.-J., Wang F., Mao C.-J., Yang Y.-P., Hu L.-F., Liu C.-F. (2017). Long-Term Levodopa Treatment Accelerates the Circadian Rhythm Dysfunction in a 6-Hydroxydopamine Rat Model of Parkinson’s Disease. Chin. Med. J..

[195] Li H., Song S., Wang Y., Huang C., Zhang F., Liu J., Hong J.-S. (2019). Low-Grade Inflammation Aggravates Rotenone Neurotoxicity and Disrupts Circadian Clock Gene Expression in Rats. Neurotox. Res..

[196] Liu W., Wei S., Huang G., Liu L., Gu C., Shen Y., Wang X., Xia S., Xie A., Hu L. (2020). BMAL1 Regulation of Microglia-mediated Neuroinflammation in MPTP-induced Parkinson’s Disease Mouse Model. FASEB J..

[197] Kim J., Jang S., Choi M., Chung S., Choe Y., Choe H.K., Son G.H., Rhee K., Kim K. (2018). Abrogation of the Circadian Nuclear Receptor REV-ERBα Exacerbates 6-Hydroxydopamine-Induced Dopaminergic Neurodegeneration. Mol. Cells.

[198] Shkodina A.D., Tan S.C., Hasan M.M., Abdelgawad M., Chopra H., Bilal M., Boiko D.I., Tarianyk K.A., Alexiou A. (2022). Roles of Clock Genes in the Pathogenesis of Parkinson’s Disease. Ageing Res. Rev..

[199] de Goede P., Wefers J., Brombacher E.C., Schrauwen P., Kalsbeek A. (2018). Circadian Rhythms in Mitochondrial Respiration. J. Mol. Endocrinol..

[200] Jacobi D., Liu S., Burkewitz K., Kory N., Knudsen N.H., Alexander R.K., Unluturk U., Li X., Kong X., Hyde A. (2015). Hepatic Bmal1 Regulates Rhythmic Mitochondrial Dynamics and Promotes Metabolic Fitness. Cell Metab..

[201] Schmitt K., Grimm A., Dallmann R., Oettinghaus B., Restelli L.M., Witzig M., Ishihara N., Mihara K., Ripperger J.A., Albrecht U. (2018). Circadian Control of DRP1 Activity Regulates Mitochondrial Dynamics and Bioenergetics. Cell Metab..

[202] Rabinovich-Nikitin I., Rasouli M., Reitz C.J., Posen I., Margulets V., Dhingra R., Khatua T.N., Thliveris J.A., Martino T.A., Kirshenbaum L.A. (2021). Mitochondrial Autophagy and Cell Survival Is Regulated by the Circadian *Clock* Gene in Cardiac Myocytes during Ischemic Stress. Autophagy.

[203] Galmozzi A., Mitro N., Ferrari A., Gers E., Gilardi F., Godio C., Cermenati G., Gualerzi A., Donetti E., Rotili D. (2013). Inhibition of Class I Histone Deacetylases Unveils a Mitochondrial Signature and Enhances Oxidative Metabolism in Skeletal Muscle and Adipose Tissue. Diabetes.

[204] Sengupta S., Yang G., O’Donnell J.C., Hinson M.D., McCormack S.E., Falk M.J., La P., Robinson M.B., Williams M.L., Yohannes M.T. (2016). The Circadian Gene Rev-Erbα Improves Cellular Bioenergetics and Provides Preconditioning for Protection against Oxidative Stress. Free Radic. Biol. Med..

[205] Newman J.C., Verdin E. (2017). β-Hydroxybutyrate. Annu. Rev. Nutr..

[206] Shen R.-S., Abell C.W., Gessner W., Brossi A. (1985). Serotonergic Conversion of MPTP and Dopaminergic Accumulation of MPP^+^. FEBS Lett..

[207] Ramsay R.R., Dadgar J., Trevor A., Singer T.P. (1986). Energy-Driven Uptake of N-Methyl-4-Phenylpyridine by Brain Mitochondria Mediates the Neurotoxicity of MPTP. Life Sci..

[208] Devi L., Raghavendran V., Prabhu B.M., Avadhani N.G., Anandatheerthavarada H.K. (2008). Mitochondrial Import and Accumulation of α-Synuclein Impair Complex I in Human Dopaminergic Neuronal Cultures and Parkinson Disease Brain. J. Biol. Chem..

[209] Cassarino D.S., Parks J.K., Parker W.D., Bennett J.P. (1999). The Parkinsonian Neurotoxin MPP+ Opens the Mitochondrial Permeability Transition Pore and Releases Cytochrome c in Isolated Mitochondria via an Oxidative Mechanism. Biochim. Biophys. Acta Mol. Basis Dis..

[210] Klaidman L.K., Adams J.D., Leung A.C., Sam Kim S., Cadenas E. (1993). Redox Cycling of MPP+: Evidence for a New Mechanism Involving Hydride Transfer with Xanthine Oxidase, Aldehyde Dehydrogenase, and Lipoamide Dehydrogenase. Free. Radic. Biol. Med..

[211] Adams J.D., Klaidman L.K., Leung A.C. (1993). MPP+ and MPDP+ Induced Oxygen Radical Formation with Mitochondrial Enzymes. Free Radic. Biol. Med..

[212] Makino K., Hagiwara T., Murakami A. (1991). A Mini Review: Fundamental Aspects of Spin Trapping with DMPO. Int. J. Radiat. Appl. Instrum. Part C Radiat. Phys. Chem..

[213] Hoehne M.N., Jacobs L.J.H.C., Lapacz K.J., Calabrese G., Murschall L.M., Marker T., Kaul H., Trifunovic A., Morgan B., Fricker M. (2022). Spatial and Temporal Control of Mitochondrial H_2_O_2_ Release in Intact Human Cells. EMBO J..

[214] Xiao B., Deng X., Lim G.G.Y., Xie S., Zhou Z.D., Lim K.-L., Tan E.-K. (2017). Superoxide Drives Progression of Parkin/PINK1-Dependent Mitophagy Following Translocation of Parkin to Mitochondria. Cell Death Dis..

[215] Zhang J., Wang B., Wang H., He H., Wu Q., Qin X., Yang X., Chen L., Xu G., Yuan Z. (2018). Disruption of the Superoxide Anions-Mitophagy Regulation Axis Mediates Copper Oxide Nanoparticles-Induced Vascular Endothelial Cell Death. Free Radic. Biol. Med..

[216] Aon M.A., Cortassa S., Marbán E., O’Rourke B. (2003). Synchronized Whole Cell Oscillations in Mitochondrial Metabolism Triggered by a Local Release of Reactive Oxygen Species in Cardiac Myocytes. J. Biol. Chem..

[217] Marchissio M.J., Francés D.E.A., Carnovale C.E., Marinelli R.A. (2012). Mitochondrial Aquaporin-8 Knockdown in Human Hepatoma HepG2 Cells Causes ROS-Induced Mitochondrial Depolarization and Loss of Viability. Toxicol. Appl. Pharmacol..

[218] Almasalmeh A., Krenc D., Wu B., Beitz E. (2014). Structural Determinants of the Hydrogen Peroxide Permeability of Aquaporins. FEBS J..

[219] Wang Y., Yen F.S., Zhu X.G., Timson R.C., Weber R., Xing C., Liu Y., Allwein B., Luo H., Yeh H.-W. (2021). SLC25A39 Is Necessary for Mitochondrial Glutathione Import in Mammalian Cells. Nature.

[220] Gialluisi A., Reccia M.G., Modugno N., Nutile T., Lombardi A., Di Giovannantonio L.G., Pietracupa S., Ruggiero D., Scala S., Gambardella S. (2021). Identification of Sixteen Novel Candidate Genes for Late Onset Parkinson’s Disease. Mol. Neurodegener..

[221] Francisco A., Engel D.F., Figueira T.R., Rogério F., de Bem A.F., Castilho R.F. (2020). Mitochondrial NAD(P)^+^ Transhydrogenase Is Unevenly Distributed in Different Brain Regions, and Its Loss Causes Depressive-like Behavior and Motor Dysfunction in Mice. Neuroscience.

[222] Palacios-Callender M., Quintero M., Hollis V.S., Springett R.J., Moncada S. (2004). Endogenous NO Regulates Superoxide Production at Low Oxygen Concentrations by Modifying the Redox State of Cytochrome c Oxidase. Proc. Natl. Acad. Sci. USA.

[223] Brown G.C. (1999). Nitric Oxide and Mitochondrial Respiration. Biochim. Biophys. Acta Bioenerg..

[224] Heck D., Kagan V., Shvedova A., Laskin J. (2005). An Epigrammatic (Abridged) Recounting of the Myriad Tales of Astonishing Deeds and Dire Consequences Pertaining to Nitric Oxide and Reactive Oxygen Species in Mitochondria with an Ancillary Missive Concerning the Origins of Apoptosis. Toxicology.

[225] MacMillan-Crow L.A., Thompson J.A. (1999). Tyrosine Modifications and Inactivation of Active Site Manganese Superoxide Dismutase Mutant (Y34F) by Peroxynitrite. Arch. Biochem. Biophys..

[226] Radi R., Cassina A., Hodara R., Quijano C., Castro L. (2002). Peroxynitrite Reactions and Formation in Mitochondria. Free. Radic. Biol. Med..

[227] Forsmark-Andrée P., Persson B., Radi R., Dallner G., Ernster L. (1996). Oxidative Modification of Nicotinamide Nucleotide Transhydrogenase in Submitochondrial Particles: Effect of Endogenous Ubiquinol. Arch. Biochem. Biophys..

[228] Zago E.B., Castilho R.F., Vercesi A.E. (2000). The Redox State of Endogenous Pyridine Nucleotides Can Determine Both the Degree of Mitochondrial Oxidative Stress and the Solute Selectivity of the Permeability Transition Pore. FEBS Lett..

[229] Ronchi J.A., Figueira T.R., Ravagnani F.G., Oliveira H.C.F., Vercesi A.E., Castilho R.F. (2013). A Spontaneous Mutation in the Nicotinamide Nucleotide Transhydrogenase Gene of C57BL/6J Mice Results in Mitochondrial Redox Abnormalities. Free. Radic. Biol. Med..

[230] Thomas M.H., Karout M., Rodriguez B.P., Gui Y., Jaeger C., Michelucci A., Kollmus H., Schughart K., Coowar D., Balling R. (2020). Strain- and Age-Dependent Features of the Nigro-Striatal Circuit in Three Common Laboratory Mouse Strains, C57BL/6J, A/J, and DBA/2J—Implications for Parkinson’s Disease Modeling. bioRxiv.

[231] Chen L., Zhang Z., Hoshino A., Zheng H.D., Morley M., Arany Z., Rabinowitz J.D. (2019). NADPH Production by the Oxidative Pentose-Phosphate Pathway Supports Folate Metabolism. Nat. Metab..

[232] Willems P.H.G.M., Rossignol R., Dieteren C.E.J., Murphy M.P., Koopman W.J.H. (2015). Redox Homeostasis and Mitochondrial Dynamics. Cell Metab..

[233] Terešak P., Lapao A., Subic N., Boya P., Elazar Z., Simonsen A. (2022). Regulation of PRKN-Independent Mitophagy. Autophagy.

[234] Lopes da Fonseca T., Villar-Piqué A., Outeiro T.F. (2015). The Interplay between Alpha-Synuclein Clearance and Spreading. Biomolecules.

[235] Peth A., Nathan J.A., Goldberg A.L. (2013). The ATP Costs and Time Required to Degrade Ubiquitinated Proteins by the 26 S Proteasome. J. Biol. Chem..

[236] Peth A., Uchiki T., Goldberg A.L. (2010). ATP-Dependent Steps in the Binding of Ubiquitin Conjugates to the 26S Proteasome That Commit to Degradation. Mol. Cell.

[237] Singh R., Cuervo A.M. (2011). Autophagy in the Cellular Energetic Balance. Cell Metab..

[238] Bell S.M., Burgess T., Lee J., Blackburn D.J., Allen S.P., Mortiboys H. (2020). Peripheral Glycolysis in Neurodegenerative Diseases. Int. J. Mol. Sci..

[239] Beg M., Abdullah N., Thowfeik F.S., Altorki N.K., McGraw T.E. (2017). Distinct Akt Phosphorylation States Are Required for Insulin Regulated Glut4 and Glut1-Mediated Glucose Uptake. eLife.

[240] Cai R., Zhang Y., Simmering J.E., Schultz J.L., Li Y., Fernandez-Carasa I., Consiglio A., Raya A., Polgreen P.M., Narayanan N.S. (2019). Enhancing Glycolysis Attenuates Parkinson’s Disease Progression in Models and Clinical Databases. J. Clin. Investig..

[241] Rhea E.M., Banks W.A. (2021). Interactions of Lipids, Lipoproteins, and Apolipoproteins with the Blood-Brain Barrier. Pharm. Res..

[242] Panov A., Orynbayeva Z., Vavilin V., Lyakhovich V. (2014). Fatty Acids in Energy Metabolism of the Central Nervous System. Biomed Res. Int..

[243] Barber C.N., Raben D.M. (2019). Lipid Metabolism Crosstalk in the Brain: Glia and Neurons. Front. Cell Neurosci..

[244] Ioannou M.S., Jackson J., Sheu S.-H., Chang C.-L., Weigel A.V., Liu H., Pasolli H.A., Xu C.S., Pang S., Matthies D. (2019). Neuron-Astrocyte Metabolic Coupling Protects against Activity-Induced Fatty Acid Toxicity. Cell.

[245] Dedkova E.N., Blatter L.A. (2014). Role of β-Hydroxybutyrate, Its Polymer Poly-β-Hydroxybutyrate and Inorganic Polyphosphate in Mammalian Health and Disease. Front. Physiol..

[246] Koronowski K.B., Greco C.M., Huang H., Kim J.-K., Fribourgh J.L., Crosby P., Mathur L., Ren X., Partch C.L., Jang C. (2021). Ketogenesis Impact on Liver Metabolism Revealed by Proteomics of Lysine β-Hydroxybutyrylation. Cell Rep..

[247] Terranova C.J., Stemler K.M., Barrodia P., Jeter-Jones S.L., Ge Z., de la Cruz Bonilla M., Raman A., Cheng C.-W., Allton K.L., Arslan E. (2021). Reprogramming of H3K9bhb at Regulatory Elements Is a Key Feature of Fasting in the Small Intestine. Cell Rep..

[248] Benderdour M., Charron G., deBlois D., Comte B., Rosiers C.D. (2003). Cardiac Mitochondrial NADP+-Isocitrate Dehydrogenase Is Inactivated through 4-Hydroxynonenal Adduct Formation: An Event that Precedes Hypertrophy Development. J. Biol. Chem..

[249] Perez M.A., Magtanong L., Dixon S.J., Watts J.L. (2020). Dietary Lipids Induce Ferroptosis in Caenorhabditis Elegans and Human Cancer Cells. Dev. Cell.

[250] Yamada N., Karasawa T., Kimura H., Watanabe S., Komada T., Kamata R., Sampilvanjil A., Ito J., Nakagawa K., Kuwata H. (2020). Ferroptosis Driven by Radical Oxidation of N-6 Polyunsaturated Fatty Acids Mediates Acetaminophen-Induced Acute Liver Failure. Cell Death Dis..

[251] Tripathi A., Fanning S., Dettmer U. (2021). Lipotoxicity Downstream of α-Synuclein Imbalance: A Relevant Pathomechanism in Synucleinopathies?. Biomolecules.

[252] Kagan V.E., Tyurin V.A., Jiang J., Tyurina Y.Y., Ritov V.B., Amoscato A.A., Osipov A.N., Belikova N.A., Kapralov A.A., Kini V. (2005). Cytochrome c Acts as a Cardiolipin Oxygenase Required for Release of Proapoptotic Factors. Nat. Chem. Biol..

[253] Jenkins C.M., Yang K., Liu G., Moon S.H., Dilthey B.G., Gross R.W. (2018). Cytochrome c Is an Oxidative Stress–Activated Plasmalogenase That Cleaves Plasmenylcholine and Plasmenylethanolamine at the Sn-1 Vinyl Ether Linkage. J. Biol. Chem..

[254] Hsu Y.-H., Dumlao D.S., Cao J., Dennis E.A. (2013). Assessing Phospholipase A2 Activity toward Cardiolipin by Mass Spectrometry. PLoS ONE.

[255] Mancuso D.J., Kotzbauer P., Wozniak D.F., Sims H.F., Jenkins C.M., Guan S., Han X., Yang K., Sun G., Malik I. (2009). Genetic Ablation of Calcium-Independent Phospholipase A2γ Leads to Alterations in Hippocampal Cardiolipin Content and Molecular Species Distribution, Mitochondrial Degeneration, Autophagy, and Cognitive Dysfunction. J. Biol. Chem..

[256] Mahajan M., Bharambe N., Shang Y., Lu B., Mandal A., Madan Mohan P., Wang R., Boatz J.C., Manuel Martinez Galvez J., Shnyrova A.V. (2021). NMR Identification of a Conserved Drp1 Cardiolipin-Binding Motif Essential for Stress-Induced Mitochondrial Fission. Proc. Natl. Acad. Sci. USA.

[257] Chu C.T., Ji J., Dagda R.K., Jiang J.F., Tyurina Y.Y., Kapralov A.A., Tyurin V.A., Yanamala N., Shrivastava I.H., Mohammadyani D. (2013). Cardiolipin Externalization to the Outer Mitochondrial Membrane Acts as an Elimination Signal for Mitophagy in Neuronal Cells. Nat. Cell Biol..

[258] Iyer S.S., He Q., Janczy J.R., Elliott E.I., Zhong Z., Olivier A.K., Sadler J.J., Knepper-Adrian V., Han R., Qiao L. (2013). Mitochondrial Cardiolipin Is Required for Nlrp3 Inflammasome Activation. Immunity.

[259] Monteiro-Cardoso V.F., Oliveira M.M., Melo T., Domingues M.R.M., Moreira P.I., Ferreiro E., Peixoto F., Videira R.A. (2015). Cardiolipin Profile Changes Are Associated to the Early Synaptic Mitochondrial Dysfunction in Alzheimer’s Disease. J. Alzheimer’s Dis..

[260] Kimura T., Kimura A.K., Ren M., Monteiro V., Xu Y., Berno B., Schlame M., Epand R.M. (2019). Plasmalogen Loss Caused by Remodeling Deficiency in Mitochondria. Life Sci. Alliance.

[261] Senanayake V., Goodenowe D.B. (2019). Plasmalogen Deficiency and Neuropathology in Alzheimer’s Disease: Causation or Coincidence?. Alzheimers Dement..

[262] Wood P.L., Smith T., Lane N., Khan M.A., Ehrmantraut G., Goodenowe D.B. (2011). Oral Bioavailability of the Ether Lipid Plasmalogen Precursor, PPI-1011, in the Rabbit: A New Therapeutic Strategy for Alzheimer’s Disease. Lipids Health Dis..

[263] Mawatari S., Ohara S., Taniwaki Y., Tsuboi Y., Maruyama T., Fujino T. (2020). Improvement of Blood Plasmalogens and Clinical Symptoms in Parkinson’s Disease by Oral Administration of Ether Phospholipids: A Preliminary Report. Parkinson’s Dis..

[264] Dorninger F., Forss-Petter S., Wimmer I., Berger J. (2020). Plasmalogens, Platelet-Activating Factor and beyond—Ether Lipids in Signaling and Neurodegeneration. Neurobiol. Dis..

[265] Jo D.S., Cho D.-H. (2019). Peroxisomal Dysfunction in Neurodegenerative Diseases. Arch. Pharm. Res..

[266] Kim J., Bai H. (2022). Peroxisomal Stress Response and Inter-Organelle Communication in Cellular Homeostasis and Aging. Antioxidants.

[267] Salem N., Litman B., Kim H.-Y., Gawrisch K. (2001). Mechanisms of Action of Docosahexaenoic Acid in the Nervous System. Lipids.

[268] Hoffman-Kuczynski B., Reo N.V. (2005). Administration of Myo-Inositol Plus Ethanolamine Elevates Phosphatidylethanolamine Plasmalogen in the Rat Cerebellum. Neurochem. Res..

[269] Torres S., García-Ruiz C.M., Fernandez-Checa J.C. (2019). Mitochondrial Cholesterol in Alzheimer’s Disease and Niemann–Pick Type C Disease. Front. Neurol..

[270] Jayashankar V., Selwan E., Hancock S.E., Verlande A., Goodson M.O., Eckenstein K.H., Milinkeviciute G., Hoover B.M., Chen B., Fleischman A.G. (2021). Drug-like Sphingolipid SH-BC-893 Opposes Ceramide-induced Mitochondrial Fission and Corrects Diet-induced Obesity. EMBO Mol. Med..

[271] Brekk O.R., Honey J.R., Lee S., Hallett P.J., Isacson O. (2020). Cell Type-Specific Lipid Storage Changes in Parkinson’s Disease Patient Brains Are Recapitulated by Experimental Glycolipid Disturbance. Proc. Natl. Acad. Sci. USA.

[272] Zhu J., Schwörer S., Berisa M., Kyung Y.J., Ryu K.W., Yi J., Jiang X., Cross J.R., Thompson C.B. (2021). Mitochondrial NADP(H) Generation Is Essential for Proline Biosynthesis. Science.

[273] Abraham M.A., Lam T.K.T. (2016). Glucagon Action in the Brain. Diabetologia.

[274] Berwick D.C., Hers I., Heesom K.J., Moule S.K., Tavareá J.M. (2002). The Identification of ATP-Citrate Lyase as a Protein Kinase B (Akt) Substrate in Primary Adipocytes. J. Biol. Chem..

[275] Brownsey R.W., Boone A.N., Elliott J.E., Kulpa J.E., Lee W.M. (2006). Regulation of Acetyl-CoA Carboxylase. Biochem. Soc. Trans..

[276] Hoxhaj G., Ben-Sahra I., Lockwood S.E., Timson R.C., Byles V., Henning G.T., Gao P., Selfors L.M., Asara J.M., Manning B.D. (2019). Direct Stimulation of NADP^+^ Synthesis through Akt-Mediated Phosphorylation of NAD Kinase. Science.

[277] Zhang Y., Xu Y., Lu W., Li J., Yu S., Brown E.J., Stanger B.Z., Rabinowitz J.D., Yang X. (2022). G6PD-Mediated Increase in De Novo NADP+ Biosynthesis Promotes Antioxidant Defense and Tumor Metastasis. Sci. Adv..

[278] Peng I.-C., Chen Z., Sun W., Li Y.-S., Marin T.L., Hsu P.-H., Su M.-I., Cui X., Pan S., Lytle C.Y. (2012). Glucagon Regulates ACC Activity in Adipocytes through the CAMKKβ/AMPK Pathway. Am. J. Physiol. Endocrinol. Metab..

[279] Wang H., Dou S., Zhu J., Shao Z., Wang C., Xu X., Cheng B. (2021). Ghrelin Protects against Rotenone-Induced Cytotoxicity: Involvement of Mitophagy and the AMPK/SIRT1/PGC1α Pathway. Neuropeptides.

[280] Kobilo T., Guerrieri D., Zhang Y., Collica S.C., Becker K.G., van Praag H. (2014). AMPK Agonist AICAR Improves Cognition and Motor Coordination in Young and Aged Mice. Learn. Mem..

[281] Ramamurthy S., Chang E., Cao Y., Zhu J., Ronnett G.V. (2014). AMPK Activation Regulates Neuronal Structure in Developing Hippocampal Neurons. Neuroscience.

[282] McCarthy C.G., Waigi E.W., Yeoh B.S., Mell B., Vijay-Kumar M., Wenceslau C.F., Joe B. (2022). Low-Dose 1,3-Butanediol Reverses Age-Associated Vascular Dysfunction Independent of Ketone Body β-Hydroxybutyrate. Am. J. Physiol. Heart Circ. Physiol..

[283] McReynolds M.R., Chellappa K., Chiles E., Jankowski C., Shen Y., Chen L., Descamps H.C., Mukherjee S., Bhat Y.R., Lingala S.R. (2021). NAD+ Flux Is Maintained in Aged Mice despite Lower Tissue Concentrations. Cell Syst..

[284] Merrill D.K., Guynn R.W. (1981). The Calculation of the Cytoplasmic Free [NADP+]/[NADPH] Ratio in Brain: Effect of Electroconvulsive Seizure. Brain Res..

[285] Parihar M.S., Kunz E.A., Brewer G.J. (2008). Age-Related Decreases in NAD(P)H and Glutathione Cause Redox Declines before ATP Loss during Glutamate Treatment of Hippocampal Neurons. J. Neurosci. Res..

[286] Ghosh D., Levault K.R., Brewer G.J. (2014). Relative Importance of Redox Buffers GSH and NAD(P)H in Age-Related Neurodegeneration and Alzheimer Disease-like Mouse Neurons. Aging Cell.

[287] Ghosh D., LeVault K.R., Barnett A.J., Brewer G.J. (2012). A Reversible Early Oxidized Redox State That Precedes Macromolecular ROS Damage in Aging Nontransgenic and 3xTg-AD Mouse Neurons. J. Neurosci..

[288] Blacker T.S., Duchen M.R. (2016). Investigating Mitochondrial Redox State Using NADH and NADPH Autofluorescence. Free Radic. Biol. Med..

[289] Subrahmanian N., LaVoie M.J. (2021). Is There a Special Relationship between Complex I Activity and Nigral Neuronal Loss in Parkinson’s Disease? A Critical Reappraisal. Brain Res..

[290] Borst P. (2020). The Malate–Aspartate Shuttle (Borst Cycle): How It Started and Developed into a Major Metabolic Pathway. IUBMB Life.

[291] Ren X., Chen J.-F. (2020). Caffeine and Parkinson’s Disease: Multiple Benefits and Emerging Mechanisms. Front. Neurosci..

[292] Zhang Q., Wu Y., Zhang P., Sha H., Jia J., Hu Y., Zhu J. (2012). Exercise Induces Mitochondrial Biogenesis after Brain Ischemia in Rats. Neuroscience.

[293] Park J., Kim J., Mikami T. (2021). Exercise-Induced Lactate Release Mediates Mitochondrial Biogenesis in the Hippocampus of Mice via Monocarboxylate Transporters. Front. Physiol..

[294] Yanagi S., Sato T., Kangawa K., Nakazato M. (2018). The Homeostatic Force of Ghrelin. Cell Metab..

[295] Liu C., Xue Y., Liu M.-F., Wang Y., Chen L. (2020). Orexin and Parkinson’s Disease: A Protective Neuropeptide with Therapeutic Potential. Neurochem. Int..

[296] Elamin M., Ruskin D.N., Masino S.A., Sacchetti P. (2017). Ketone-Based Metabolic Therapy: Is Increased NAD+ a Primary Mechanism?. Front. Mol. Neurosci..

[297] Izuta Y., Imada T., Hisamura R., Oonishi E., Nakamura S., Inagaki E., Ito M., Soga T., Tsubota K. (2018). Ketone Body 3-hydroxybutyrate Mimics Calorie Restriction via the Nrf2 Activator, Fumarate, in the Retina. Aging Cell.

[298] Youm Y.-H., Nguyen K.Y., Grant R.W., Goldberg E.L., Bodogai M., Kim D., D’Agostino D., Planavsky N., Lupfer C., Kanneganti T.D. (2015). Ketone Body β-Hydroxybutyrate Blocks the NLRP3 Inflammasome-Mediated Inflammatory Disease. Nat. Med..

